# Proteomic Profiling of Bronchoalveolar Lavage Fluid in Critically Ill Patients with Ventilator-Associated Pneumonia

**DOI:** 10.1371/journal.pone.0058782

**Published:** 2013-03-07

**Authors:** Elizabeth V. Nguyen, Sina A. Gharib, Steven J. Palazzo, Yu-hua Chow, David R. Goodlett, Lynn M. Schnapp

**Affiliations:** 1 Department of Medicinal Chemistry, University of Washington, Seattle, Washington, United States of America; 2 Center for Lung Biology, Division of Pulmonary and Critical Care Medicine, Department of Medicine, University of Washington, Seattle, Washington, United States of America; 3 College of Nursing, Seattle University, Seattle, Washington, United States of America; University of Giessen Lung Center, Germany

## Abstract

**Rationale:**

Ventilator-associated pneumonia (VAP) is a common complication in patients with acute lung injury (ALI) and can lead to increased morbidity and mortality. Identifying protein profiles specific to VAP in bronchoalveolar lavage fluid (BALF) may aid in earlier diagnosis, elucidate mechanisms of disease, and identify putative targets for therapeutic intervention.

**Methods:**

BALF was obtained from 5 normal subjects and 30 ALI patients: 14 with VAP (VAP^+^) and 16 without VAP (VAP^–^). Each sample underwent shotgun proteomic analysis based on tandem mass spectrometry. Differentially expressed proteins between the groups were identified using statistical methods based on spectral counting. Mechanisms of disease were explored using functional annotation and protein interaction network analysis. Supervised classification algorithms were implemented to discover a proteomic classifier for identifying critically ill patients with VAP.

**Results:**

ALI patients had distinct BALF proteomic profiles compared to normal controls. Within the ALI group, we identified 76 differentially expressed proteins between VAP^+^ and VAP^–^. Functional analysis of these proteins suggested activation of pro-inflammatory pathways during VAP. We identified and validated a limited proteomic signature that discriminated VAP^+^ from VAP^–^ patients comprised of three proteins: S100A8, lactotransferrin (LTF), and actinin 1 (ACTN1).

**Conclusions:**

Combining proteomic with computational analyses is a powerful approach to study the BALF proteome during lung injury and development of VAP. This integrative methodology is a promising strategy to differentiate clinically relevant subsets of ALI patients, including those suffering from VAP.

## Introduction

Ventilator-associated pneumonia (VAP), defined as pneumonia occurring after 48 hours of intubation and mechanical ventilation, is a major cause of increased mortality and morbidity in critically ill patients [1]. Clinical diagnosis of VAP is especially challenging in the setting of acute lung injury (ALI) since many signs and symptoms of pneumonia such as fever, abnormal radiographs, and elevated white blood cell counts are common in patients with ALI. Culturing samples from airways using bronchoscopy with lavage or protected brushings is a key step in identifying VAP, but can also delay diagnostic and treatment plans. Therefore, identifying biomarkers for VAP in serum or bronchoalveolar lavage fluid (BALF) may provide a rapid, accurate and clinically useful test for early detection and risk stratification of critically ill patients [2]. A number of individual proteins have been proposed as biomarkers for the presence of VAP, but single biochemical measurements are not consistent predictors of either onset or severity of VAP. Serum procalcitonin [3], serum C-reactive protein [4], and BALF soluble triggering receptor expressed on myeloid cells-1 (sTREM-1) [5] are several proposed VAP candidates. However, predictive and quantitative threshold levels for these single biomarkers of VAP have been disappointingly nonspecific when follow-up validation studies were attempted [6], [7].

We hypothesized that comprehensively profiling the proteomic landscape of BALF in ALI patients would identify distinct protein signatures that could be useful as diagnostic classifiers for VAP, while providing insights into the pathogenesis of this complex disorder. To this end, we integrated tandem mass spectrometry-based proteomics with statistical and computational methods to identify differentially expressed proteins between patients with and without VAP (VAP^+^, VAP^–^). Using classification algorithms, we narrowed our candidate list and confirmed its predictive value in an independent cohort of critically ill patients undergoing evaluation for clinical suspicion of VAP.

## Materials and Methods

### Patient Recruitment and BALF Collection

The protocol for collecting human BALF was approved by the Institutional Review board at the University of Washington. Patients with ALI undergoing bronchoscopy for clinically suspected VAP were enrolled. Written informed consent was obtained from patients or a legal next of kin relative. ALI was defined using the following criteria: 1) PaO_2_/FiO_2_ <200 mm Hg on ≥5 cm of H_2_O positive end-expiratory pressure, 2) diffuse parenchymal infiltrates, 3) pulmonary artery wedge pressure <18 mm Hg or lack of clinical evidence of congestive heart failure, and 4) no other obvious explanation for these findings. Clinical criteria for suspected VAP included: 48 hours of mechanical ventilation, new or progressive pulmonary infiltrates on radiograph, and one or more of the following: fever, leukocytosis or leukopenia, an increase in purulent endotracheal secretions, and no change in antibiotics for 72 hours. Normal, healthy volunteers (n = 5) and ALI subjects (n = 30) had BALF collected as previously described [8], [9]. Briefly, five separate 30-mL aliquots of 0.89% sterile saline were instilled into the right middle lobe or lingula of left lung. Immediately on collection BALF was centrifuged at 14,000 g for 20 minutes at 4°C and cell-free supernatants aliquoted and stored at −80°C. Total protein measurements were determined on aliquots of supernatants using the Bicinchoninic acid (BCA) protein assay. Clinically suspected VAP was diagnosed when a bacterial cell culture of ≥10,000 colony forming units (cfu) per milliliter (ml) or protected specimen brush culture ≥1,000 cfu/ml was grown. Subject and BALF sample characteristics are shown in Table 1. For more comprehensive patient characteristics see Table S1.

**Table 1:** Characteristics of ALI patients.

Subject characteristics	VAP^+^ (n = 14)	VAP^–^ (n = 16)	*P*-value†
Age (mean ± SD)	53±31	48±28	0.64
Gender	14 male, 0 female	10 male, 6 female	
Ventilator days* (mean ± SD)	10±25	15±18	0.53
P_a_O_2_/F_i_O_2_ ** (mean ± SD)	206±54 mmHg	212±48 mmHg	0.75

* Number of days on the ventilator prior to bronchoscopy.

** Ratio of arterial oxygen partial pressure (P_a_O_2_) to fraction of inspired oxygen (F_i_O_2_) at time of bronchoscopy.

† Two-tailed t-test with unequal variance.

Similar criteria and procedures were used to recruit and obtain BALF from a separate group of mechanically ventilated patients suspected of having VAP for validation of selected BALF proteins (Table S2).

### Shotgun Proteomics Analysis

Tryptic digests of each BALF sample were analyzed by HPLC-MS/MS using a NanoAquity HPLC system (Milford, MA, USA) via electrospray ionization on-line to a hybrid linear ion trap-orbitrap mass spectrometer (Thermo Fisher, San Jose, CA, USA). The normal BALF samples were analyzed in an identical manner on a hybrid LTQ-Velos mass spectrometer (Thermo Fisher, San Jose, CA, USA). The experiment was repeated in triplicate using gas phase fraction over the following three *m/z* ranges: 400–559, 559–846, 846–2000 [10]. Tandem mass spectral RAW (ThermoFinnigan, Waltham, MA, USA) files were first converted to mzXML format [11]. The tandem mass spectra were then matched to a protein sequence in the IPI Human 3.53 database using SEQUEST [12]. The criteria for matching a peptide tandem mass spectrum to a peptide sequence were: X_corr_ >1.9 with charge state 1+, X_corr_ >2.22 with charge state 2+, or X_corr_ >3.75 with charge state 3+, as well as ΔCn >0.1. Peptide tandem mass spectra passing these criteria were utilized for protein identifications. A protein was considered to be identified only if ProteinProphet probability was >0.8 and more than one unique peptide was identified [13]. For each pair-wise group comparison (i.e., normal, VAP^+^, VAP^–^), we restricted analysis to those proteins that were present in at least half the subjects of one group.

### Correspondence and Cluster Analyses

Correspondence analysis, a method to produce low-dimensional projection of high dimensional data, was performed based on the variability in protein expression in normal and ALI populations [14]. We performed hierarchical clustering of differentially expressed proteins between ALI subsets (VAP^+^ vs. VAP^–^). K-medians cluster analysis was performed on the expression of the limited proteomic classifier across all 30 ALI patients [15].

### Differential Protein Expression

To assess differences in relative protein abundance between subject populations, individual protein spectral counts were normalized using the spectral index (SI) metric as previously described [16]. SI compares relative protein abundance between two groups of samples by normalizing their values to a range of [−1, +1]. For a given protein, a SI close to +1 implies abundacne in one group (e.g., VAP^+^) whereas a value close to −1 signifies abundance in the other group (e.g., VAP^–^). A value close to 0 indicates that a protein is about equally abundant between the subjects of both groups. Statistical significance is determined through a random permutation analysis (n = 10,000 permutations). We chose a 95% confidence threshold for significant differential expression.

### Functional Analysis

Functional annotation of the BALF proteomes for normal and ALI patients was conducted using database for annotation, visualization, and integrated discovery (DAVID) software [17]. Overrepresented functional categories among proteins enriched in each subject population were identified relative to the entire human proteome using a permutation-based false discovery rate analysis (FDR). Processes with at least three protein members and FDR <5% were deemed significant.

### Protein Network Analysis

We utilized experimentally verified and published protein-protein interactions from several resources including Ingenuity's knowledgebase [18] and STRING [19] to construct a relational network comprised of differentially expressed proteins in critically ill patients with VAP.

### Western Blot Analysis

For Western analysis, equal volumes of BALF samples from a set of subjects not previously used in the shotgun proteomic analysis were separated by 15% SDS-PAGE, transferred to Immobilon membrane, and blocked for 1 hour. Blots were incubated with polyclonal antibody S100A8 (Diagnostic Standards Laboratory), for 1 hour, followed by peroxidase-conjugated secondary antibody for 1 hour, and then developed with ECL. To quantify relative band intensities, gels were captured with Photoshop (Adobe Systems Inc., San Jose, CA, USA) and then imported into ImageJ (version 1.30; National Institutes of Health, USA) for analysis [20]. Difference between groups was assessed with the Mann-Whitney test (Prism5 software, Graphpad, USA).

### Elisa

Lactotransferrin levels were measured in duplicate by ELISA (MyBioSource, San Diego, CA, USA) per manufacturer's instructions. Concentrations were extrapolated from simultaneously run standard curves. Difference between the groups (VAP^+^ n = 8, VAP^–^ n = 7) was assessed with the Mann-Whitney test (Prism5 software, Graphpad, USA). Actinin 1 levels in the BALF from patients with or without VAP (n = 13 and 18, respectively) were measured by ELISA following manufacturer's instruction (MyBioSource, San Diego, CA). Because of larger sample sizes, difference between groups was assessed using Student's *t*-test (Prism5 software, Graphpad, USA).

## Results

### Overview of BALF Proteome in Healthy Subjects and Patients with ALI

**Figure 1:**
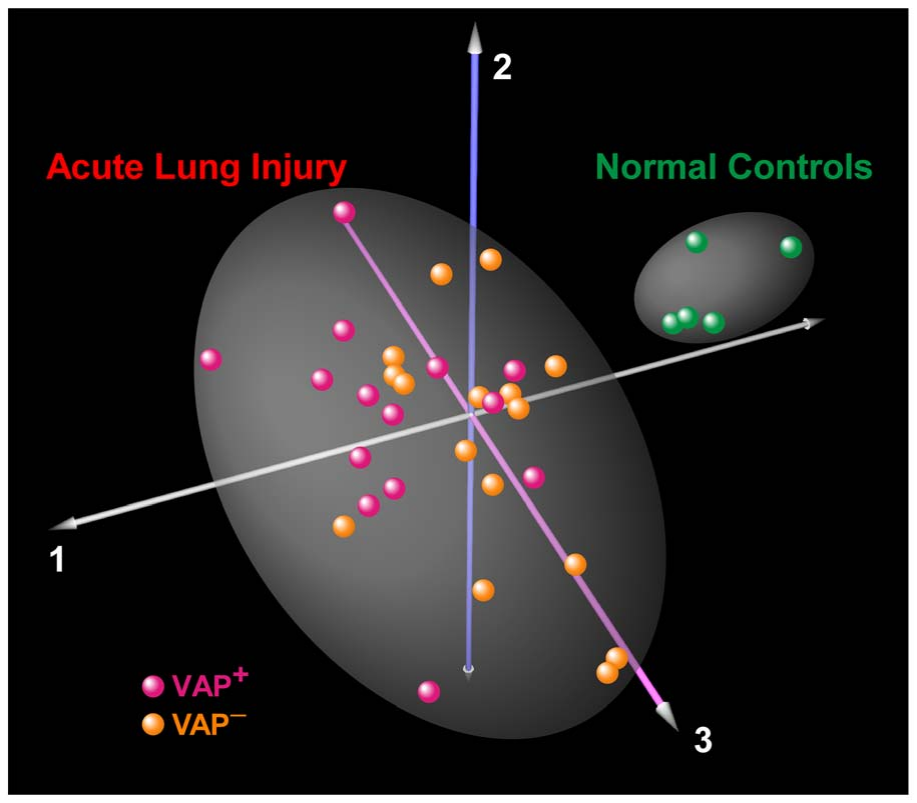
Correspondence analysis of BALF proteome in patients with ALI (n = 30) and healthy controls (n = 5). Each sphere represents an individual whose location in “proteome space” is determined by BALF protein expression variability. Each axis represents a principal coordinate that captures a component of the variance in protein expression. As expected, ALI patients segregate from the controls, implying that lung injury elicits a global perturbation in airspace protein levels. However, within the ALI group, VAP^+^ and VAP^–^ patients are not separated into distinct clusters.

We identified 1288 unique proteins in the BALF of all 35 subjects (5 normal, 14 ALI: VAP^+^, 16 ALI: VAP^–^). Many of these proteins were not detected in most subjects, suggesting very low abundance. To improve confidence and ensure biologic relevance, we restricted our analysis to proteins that were detected in greater than 50% of subjects in at least one of the subgroups. We identified 251 unique proteins in the BALF of 5 normal volunteers and 394 unique proteins in 30 ALI subjects (i.e., VAP^+^ and VAP^–^). Of these, 184 proteins were common to both groups. The most abundant protein based on raw spectral counts across all BALF samples was albumin. Tables S3 and S4 contain lists of proteins found in normal and ALI subjects. Correspondence analysis of all identified BALF proteins distinctly separated normal subjects from ALI patients, implying that lung injury causes global changes in the expression levels of proteins in the airspaces (Figure 1).

### BALF Proteome in ALI

We applied a statistical test based on protein spectral counts known as the spectral index to identify 166 differentially expressed proteins between normal and ALI subjects. Of these, 47 proteins were significantly more abundant in normal BALF, whereas 119 were differentially upregulated in the BALF of ALI patients (Table S4). To better elucidate the pathways and mechanisms activated in ALI, we performed Gene Ontology analysis on the differentially expressed BALF proteins. As shown in Table 2, the proteins upregulated during ALI mapped to a number of highly overrepresented functional categories including defense response (*P*-value: 2×10^–15^), inflammatory response (*P*-value: 5×10^–14^), wound healing (*P*-value: 7×10^–13^), immunity (*P*-value: 1×10^–9^). In contrast, downregulated proteins in ALI (and therefore more abundant in normal BALF) mapped to enriched processes involved in endopeptidase inhibitor activity (*P*-value: 3×10^–10^) and metabolic processes (*P*-value: ∼1×10^–5^).

**Table 2:** Functional analysis of differentially expressed proteins in the BALF of patients with ALI and Controls.

Enriched in BALF of ALI patients		
Gene Ontology Category	Number of Proteins	Enrichment *P*-value
Protein binding	101	2.1×10^–15^
Defense response	30	7.2×10^–15^
Acute inflammatory response	15	4.7×10^–14^
Response to wounding	26	7.2×10^–13^
Response to stress	44	1.6×10^–12^
Immune system process	30	1.1×10^–9^
Response to stimulus	59	2.6×10^–9^
Cytoskeletal protein binding	20	5.5×10^–9^
Innate immune response	11	1.8×10^–7^
Humoral immune response	9	2.5×10^–7^

Enriched in BALF of Control subjects		
Gene Ontology Category	Number of Proteins	Enrichment *P*-value
Endopeptidase inhibitor activity	10	2.8×10^–10^
Cofactor metabolic process	7	1.1×10^–5^
Enzyme regulator activity	11	1.2×10^–4^
Hexose metabolic process	6	1.4×10^–4^

### BALF Proteome in VAP

**Figure 2:**
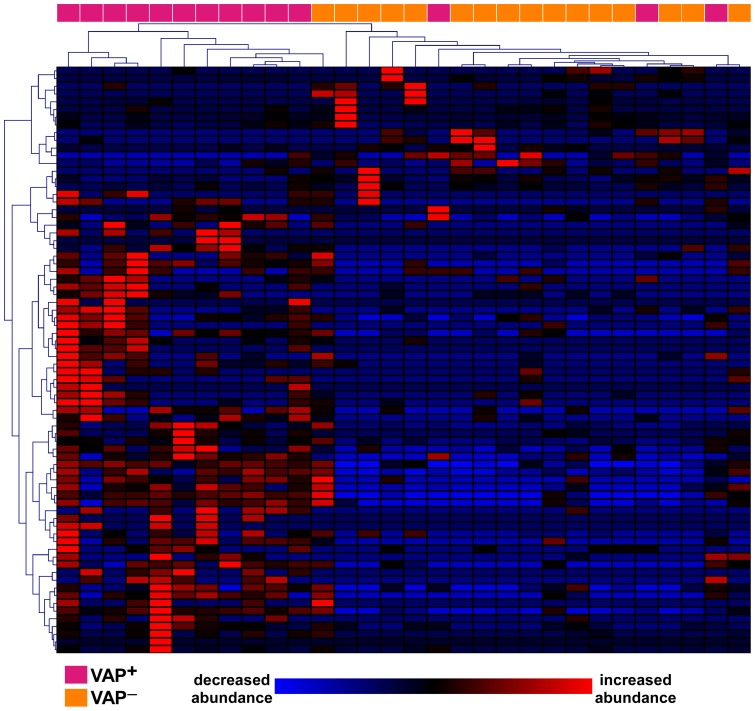
Two-dimensional hierarchical clustering of ALI patients based on the profiles of differentially expressed proteins. This unsupervised methodology demonstrates that relative abundances of 75 BALF proteins can reasonably discriminate patients with VAP from those without it.

As shown in Figure 1, correspondence analysis of the BALF proteome of all subjects did not segregate the subset of ALI patients with VAP (VAP^+^, n = 14) from those without it (VAP^–^, n = 16). This is likely due to the dominant effect of lung injury on global protein expression and variability in BALF, regardless of the presence or absence of pneumonia. Therefore, we applied the spectral index method to compare the BALF proteome between VAP^+^ and VAP^–^ patients, and identified 75 differentially expressed proteins out of a total of 394 unique proteins (Table S5). The majority of the differentially expressed proteins were upregulated in BALF of VAP^+^ subjects (n = 60 proteins), whereas 15 proteins were more abundantly expressed in VAP^–^ patients. We subsequently performed unsupervised hierarchical cluster analysis on the differentially expressed proteomic profiles of all 30 ALI patients and demonstrated that this signature is a reasonably powerful discriminator between VAP^+^ and VAP^–^ subjects (Figure 2). Functional categorization of differentially expressed proteins revealed that mechanisms involved in defense response, immunity, response to bacterium and leukocyte migration were enriched in the VAP^+^ subjects, whereas fibrinogen complex, cell surface binding, wound healing and developmental processes were overrepresented in the VAP^–^ patients (Table 3). These findings suggest that there is persistent activation of anti-bacterial and immunologic pathways in the airspace milieu of critically ill patients with VAP, whereas reparative mechanisms have been initiated in the lungs of ALI patients without VAP.

**Table 3:** Functional analysis of differentially expressed proteins in the BALF of VAP^+^ and VAP^–^ patients.

Enriched in BALF of VAP^+^ patients		
Gene Ontology Category	Number of Proteins	Enrichment *P*-value
Defense response	14	4.8×10^–7^
Protein binding	48	6.9×10^–7^
Calcium ion binding	13	9.0×10^–5^
Immune system process	14	9.1×10^–5^
Response to other organism	7	8.9×10^–4^
Defense response to bacterium	5	9.4×10^–4^
Leukocyte migration	4	1.4×10^–3^

Enriched in BALF of VAP^–^ patients		
Gene Ontology Category	Number of Proteins	Enrichment *P*-value
Fibrinogen complex	3	1.2×10^–3^
Developmental process	9	2.4×10^–3^
Cell surface binding	13	3.2×10^–3^
Wound healing	4	7.5×10^–3^

### Protein Interaction Network in VAP

**Figure 3:**
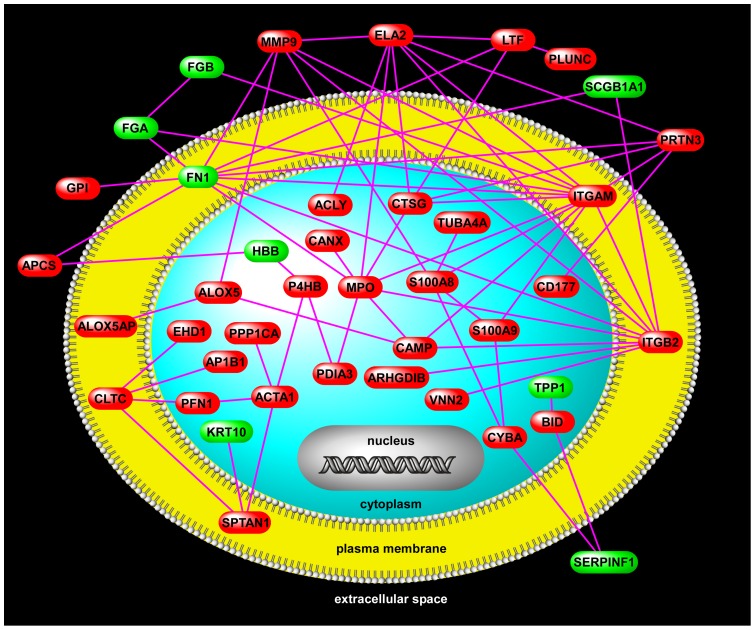
A VAP protein-protein interaction network. The members of this network are comprised of differentially up-regulated (red) and down-regulated (green) proteins in critically ill patients with VAP relative to those without VAP. Connectivity is based on previously published protein interactions. Note the presence of several densely connected hubs (ITGAM, ITGB2, MPO) that may play important roles in the pathogenesis of VAP (see Discussion). Two members of the proposed limited proteomic signature for diagnosis of VAP (S100A8 and LTF) are also network nodes.

To better elucidate functional relationships among differentially expressed proteins in VAP, we performed a network analysis using available protein-protein interaction databases (Figure 3). This putative network highlights complex partnerships between proteins that are functionally active in the extracellular space (e.g., MMP9, ELA2, LTF, PLUNC), plasma membrane (e.g., ITGAM, ITGB2), and the cytoplasm (e.g., S100A8, S100A9, MPO, CTSG).

### Development and Validation of a Proteomic Classifier for VAP

**Figure 4:**
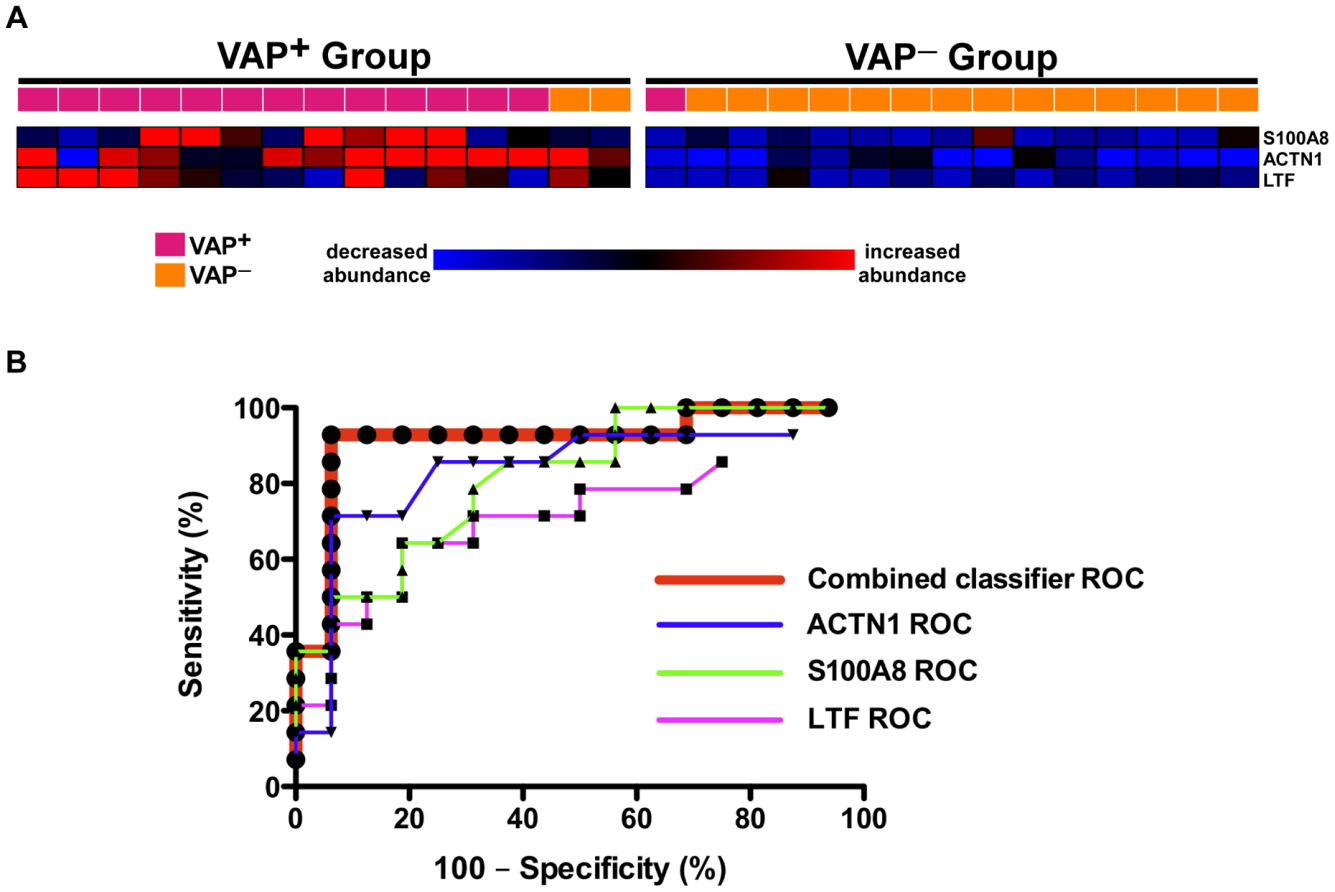
Supervised discrimination analysis integrated with the spectral index method identified a 3-protein signature that robustly segregated VAP^+^ from VAP^–^. Panel **A**: K-median clustering was performed based on the expression profiles of these 3 proteins, resulting in the correct classification of 27 out of 30 patients. Panel **B**: ROC analysis demonstrates the superior performance of the combined protein classifier compared to each individual member.

Although useful in investigating mechanisms of disease, the large number of differentially expressed proteins between VAP^+^ and VAP^–^ precludes its practical utility as a diagnostic classifier. Therefore, we employed a two-step strategy to discover a limited proteomic predictor for VAP. Initially we applied a supervised classification algorithm known as prediction analysis of microarrays (PAM) to the entire ALI BALF proteomic dataset of 394 proteins [21]. PAM was used to train and cross validate a set of proteins with robust properties to discriminate VAP^+^ and VAP^–^ patients. However, this list was comprised of 28 proteins. Since our aim was to develop a much smaller classifier, we selected the top three proteins with (i) the highest discrimination scores between the groups and (ii) spectral index values reaching 95% confidence levels. This limited proteomic predictor included S100A8, lactotransferrin (LTF), and actinin 1 (ACTN1). As shown in Figure 4A, when the 30 ALI patients underwent unsupervised K-median clustering based on the expression profiles of these 3 proteins, 90% of the subjects were correctly classified. We further assessed the performance of this classifier using receiver operating characteristic (ROC) analysis (Figure 4B). For this analysis, the spectral counts of each protein were normalized across all 30 patients and then summed per subject to obtain a combined classifier signature. This integrated discriminator significantly outperformed any of its individual member proteins, achieving an optimal sensitivity specificity profile of 93% and 94% respectively.

**Figure 5:**
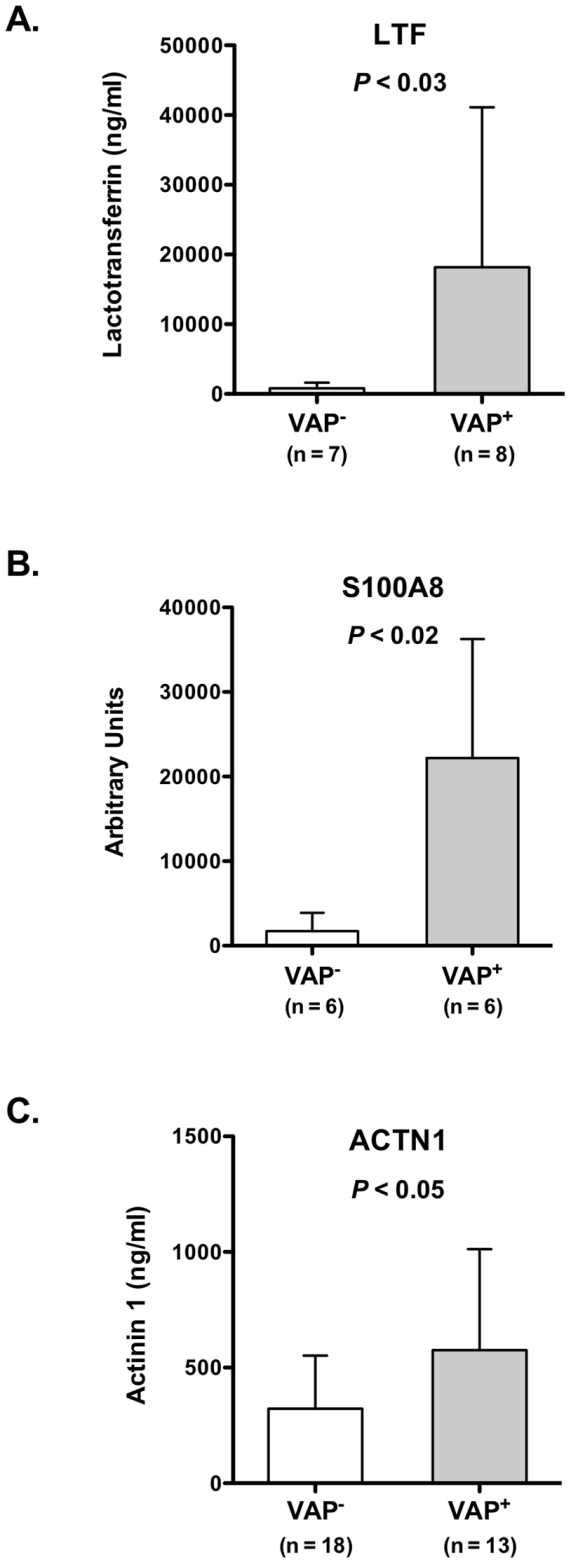
Validation of the limited proteomic classifier in an independent cohort of critically ill patients with clinical suspicion of VAP. Panel **A** confirms elevated levels of LTF in BALF of VAP^+^ patients compared to VAP^–^ subjects using ELISA. Panel **B** demonstrates increased abundance of BALF S100A8 in VAP^+^ based on Western analysis (see also supplementary Figure S1). Panel **C** displays increased levels of ACTN1 in BALF of VAP^+^ patients compared to VAP^–^ using ELISA.

Two members of this classifier, S100A8 and LTF, mapped to the VAP protein interactome (Figure 3). To validate the predictive power of the VAP proteomic signature, we biochemically assessed the levels of S100A8, LTF and ACTN1 in the BALF of a separate group of mechanically ventilated patients with clinical suspicion for VAP (Figure 5). These critically ill patients had similar clinical characteristics to our original discovery cohort (Table S2).

## Discussion

Ventilator-associated pneumonia remains a common complication in critically ill patients and adversely influences clinical outcomes. Our study represents the largest investigation of the BALF proteome in patients with acute lung injury and VAP. Using a shotgun proteomics approach, we not only identified differentially expressed proteins in the BALF of ALI subjects with VAP, but also developed a limited proteomic signature to discriminate critically ill patients with VAP from those without it. A previous report by Lu *et* *al* used a proteomics approach on BALF from five patients suspected of having VAP [22]. However the study was limited because the number of patients was small, BALF samples were pooled before proteomics analysis, and the diagnosis of VAP depended on elevated levels of sTREM-1 – a biomarker that has poor specificity for VAP [7].

In this study, we established that compared to normal individuals, the BALF proteome of ALI patients was highly enriched in proteins involved in inflammation, defense response and immunity (Table 2). However, global proteomic profiling of normal and ALI subjects did not segregate ALI subsets (VAP^+^ and VAP^–^ Figure 1), most likely because acute lung, injury (regardless of VAP status) is the dominant effector of protein expression in BALF. We therefore focused our analysis to the 30 ALI patients (14 VAP^+^ and 16 VAP^–^) and identified 76 differentially expressed proteins (Figure 2). The majority of these proteins were more abundant in the BALF of VAP^+^ patients, and mapped to distinct functional categories that included defense response and response to bacterium (Table 3) – highlighting ongoing activation of these pro-inflammatory processes. In contrast, the BALF of VAP^–^ subjects was enriched in proteins involved in wound healing and development, suggesting that in the absence of active infection, reparative programs were induced following acute lung injury.

We further explored functional relationships among differentially expressed proteins in VAP using gene product interaction network analysis (Figure 3). Complex biological networks possess topologic properties that are functionally informative [23]. For example, highly connected nodes – known as hubs, disproportionately affect the stability of a network and may be important orchestrators of biological processes [23], [24]. Figure 3 depicts several such hubs, including integrin beta 2 (ITGB2), integrin alpha M (ITGAM), and myeloperoxidase (MPO) – all of which were up-regulated in VAP^+^ patients and directly interacted with each other. The β_2_ integrins ITGB2 and ITGAM (also known as CD18 and CD11b) are critical mediators of neutrophil adhesion and transmigration across the endothelium and promote bacterial phagocytosis [25]. Their up-regulation in VAP^+^ subjects suggests activation and infiltration of leukocytes into the airspaces. Furthermore, studies demonstrate that MPO, a potent phagocyte-derived peroxidase, suppresses neutrophil apoptosis through signaling via ITGB2 and ITGAM thereby prolonging inflammation and contributing to persistent lung injury [26]. In contrast, another network hub, fibronectin 1 (FN1), was downregulated in VAP^+^ subjects. FN1, a principal component of the extracellular matrix, is a key regulator of epithelial wound repair [27]. FN1 was recently reported to be down-regulated in airway epithelial cells of asthmatics and this suppression contributed to impaired airway repair in these patients [28]. These findings suggest that VAP is characterized by an imbalance between overabundance of pro-inflammatory mediators and reduced expression of reparative proteins. Although our analysis was primarily descriptive, integrating shotgun proteomics with bioinformatics and network analysis provided unique insights into the pathogenesis of VAP in critically ill patients and identified putative mechanisms whereby VAP promotes further lung injury and delays healing processes.

Early diagnosis and treatment of VAP may reduce the morbidity and mortality in critically ill patients, but robust biomarkers have not been identified to date. The primary role of such biomarkers is to facilitate rapid clinical decision-making regarding initiation of antimicrobial therapy before a definitive diagnosis is established via quantitative cultures. Although this study was not designed or powered to validate biomarkers using large clinical cohorts, a novel feature of our shotgun proteomics approach was its amenability for supervised discriminatory analysis between phenotypes. Using a two-step approach, we identified a limited proteomic signature that accurately separated VAP^+^ and VAP^–^ patients (Figure 4). We validated the robustness of the classifier by prospectively measuring two member proteins, lactotransferrin (LTF) and S100 calcium binding protein A8 (S100A8), in a group of ALI patients undergoing bronchoscopy for suspicion of VAP. We confirmed that the expression patterns of LTF and S100A8 in the validation cohort mimicked those of the original 30 ALI subjects, with both proteins being significantly more abundant in BALF of VAP^+^ patients (Figure 5). Although the utility of such signatures is solely due to their ability to distinguish classes based on distinct expression patterns, we noted that two members of the classifier (LTF and S100A8) mapped to the VAP interactome (Figure 3). This observation implied that these candidates – beyond their discriminatory properties – may also have biologic roles in the pathogenesis of VAP.

LTF is a key component of the respiratory tract antimicrobial defense system and innate immunity [29]. Elevated LTF levels have been reported in the BALF of patients with cystic fibrosis [30], chronic bronchitis [31], and acute respiratory distress syndrome [32]. S100A8 is an antimicrobial protein expressed by leukocytes and epithelial cells, often partnering with S100A9 to inhibit bacterial adhesion to epithelium and limit infectivity. S100A8 is a potent neutrophil chemoattractant and recruits these cells to sites of inflammation in response to lipopolysaccharide [33]. Animal studies have demonstrated that S100A8 plays a critical role in promoting the transepithelial migration of leukocytes into the airspaces during bacterial pneumonia, whereas its blockade dramatically diminishes this inflammatory process [34]. Therefore, our BALF proteomic signature discriminated ALI patients with VAP from those without pneumonia and identified putative effectors of disease propagation.

Our study has a number of limitations. First, our findings are based on a relatively small number of subjects and need to be confirmed independently in larger cohorts of critically ill patients at risk for developing VAP. Secondly, we did not assess changes in BALF protein levels over time, although all enrolled patients had been intubated for >48 hours. Temporal assessment may likely yield even more robust differences. Third, since all ALI patients (VAP^+^ and VAP^–^) were mechanically ventilated, we did not design our study to assess the independent contribution of mechanical ventilation. Mechanical ventilation likely plays an important role in altering the BALF proteome of ALI patients compared to the normal control group. Fourth, despite its comprehensive coverage, shotgun proteomics is still biased towards detecting proteins with larger molecular weights and higher abundance – thus, smaller proteins (such as chemokines) may not be identified using this method. Furthermore, protein identification is based on statistical models with estimated error thresholds, which can lead to incorrect protein identification. Since we do not know the relative contribution of different cell types in airspaces to the BALF proteome, our network analysis and proposed mechanisms of VAP pathogenesis must be considered speculative. Follow-up corroboration of the roles played by candidate targets in VAP is necessary. The VAP proteomic classifier identified in our study is not unique, and other statistical methodologies based on the BALF proteome may yield different yet highly informative biomarker panels. Finally, the robustness of this classifier needs to be validated in larger patient cohorts.

In conclusion, we have outlined a systematic, proteomics-based approach to segregate ALI patients with and without VAP based on differentially expressed proteins in the BALF. We identified a limited protein classifier that possessed discriminatory properties while maintaining biological relevance. Collectively, our findings suggest that integrating computational tools with proteomics of BALF is a promising venue for classifying and prognosticating pulmonary disorders, and may provide mechanistic insights into disease pathogenesis.

## Supporting Information

Figure S1 **Representative BALF Western blots for S100A8 showing increased expression in VAP^+^ patients.**
(PDF)

Table S1 **Detailed characteristics of ALI patients.**
(PDF)

Table S2 **Characteristics of patients suspected of having VAP in the validation cohort.**
(PDF)

Table S3 **BALF proteome of Control subjects with spectral counts.**
(PDF)

Table S4 **Differential BALF protein expression between Controls and ALI patients.**
(PDF)

Table S5 **BALF proteome of ALI patients with and without VAP.**
(PDF)
