# Supplementary material for: Proteomic Profiling of Bronchoalveolar Lavage Fluid in Critically Ill Patients with Ventilator-Associated Pneumonia
Source: PLoS One. 2013 Mar 7;8(3):e58782. doi: 10.1371/journal.pone.0058782 (PMC3591362; doi:10.1371/journal.pone.0058782)

Figure S1. Representative BALF Western blots for S100A8 showing increased expression in VAP+ patients. Please refer to Figure 5 in main manuscript for further results.

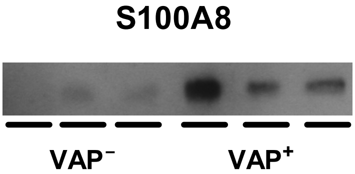

Supplement: Figure S1 — Representative BALF Western blots for S100A8 showing increased expression in VAP+ patients. (PDF) [file pone.0058782.s001.pdf]
