# Supplementary material for: Proteomic Profiling of Bronchoalveolar Lavage Fluid in Critically Ill Patients with Ventilator-Associated Pneumonia
Source: PLoS One. 2013 Mar 7;8(3):e58782. doi: 10.1371/journal.pone.0058782 (PMC3591362; doi:10.1371/journal.pone.0058782)
Supplement: Table S1 — Detailed characteristics of ALI patients. (PDF) [file pone.0058782.s002.pdf]

Table S1. Detailed characteristics of ALI patients. State of discharge, age, sex, VAP diagnosis, microbiology of bacterial strain(s) detected in culture, number of days on ventilator, P/F ratio, and risk factor provided.

Subject	State at discharge	Age	Sex	VAP	Microbiology	Vent days*	P/F**	ALI risk factor
1	Alive	60	M	Yes	*Bifidobacterium scardovii*	5/18	162	Sepsis
2	Alive	40	M	Yes	*Staphylococcus aureus, Streptococci*	2/7	185	Trauma
3	Alive	76	M	Yes	*Escherichia coli, Enterococcus*	7/11	148	Trauma
4	Alive	22	M	Yes	*Alpha streptococci, MRSA*	6/8	247	Sepsis
5	Alive	56	M	Yes	*Haemophilus influenzae*	3/13	240	Trauma
6	Alive	56	M	Yes	*Streptococcus pseudopneumoniae, Staphylococcus aureus, Haemophilus influenzae*	6/19	247	Burn, sepsis
7	Alive	25	M	Yes	*Alpha streptococcus, Diphtheroids*	19/38	280	Trauma
8	Alive	69	M	Yes	*Staphylococcus aureus*	5/13	310	Sepsis
9	Alive	25	M	Yes	*Staphylococcus aureus, Acinetobacter*	11/26	138	Trauma
10	Dead	79	M	Yes	*Pseudomonas aeruginosa*	16/35	185	Trauma
11	Alive	66	M	Yes	*Staphylococcus aureus*	14/15	173	Trauma
12	Dead	62	M	Yes	*Staphylococcus aureus*	7/9	187	Cardiogenic shock
13	Alive	46	M	Yes	*Streptococcus milleri*	4/21	141	Trauma
14	Dead	65	M	Yes	*Pseudomonas aeruginosa*	12/17	240	Sepsis
15	Alive	70	M	No	N/A	18/33	212	Trauma
16	Alive	38	F	No	N/A	5/13	144	Pancreatitis
17	Alive	22	M	No	N/A	3/5	138	Trauma
18	Alive	20	M	No	N/A	10/30	185	Trauma
19	Alive	62	F	No	N/A	7/11	254	Sepsis
20	Alive	53	M	No	N/A	4/10	223	Sepsis, shock
21	Alive	61	M	No	N/A	9/15	208	Sepsis
22	Alive	56	F	No	N/A	2/5	250	Sepsis
23	Dead	60	M	No	N/A	10/25	220	Cancer
24	Alive	62	F	No	N/A	1/10	234	Sepsis
25	Alive	49	M	No	N/A	4/11	168	Trauma
26	Alive	59	F	No	N/A	6/11	300	Trauma
27	Alive	40	M	No	N/A	11/25	300	Trauma
28	Alive	49	F	No	N/A	5/17	183	Sepsis
29	Alive	42	M	No	N/A	5/35	170	Burns
30	Alive	25	M	No	N/A	13/38	203	Trauma

* Days on ventilator prior to bronchoscopy/total number of days on the ventilator
**P/F ratio closest to time of bronchoscopy (all patients had P/F < 300 at time of ALI dx)
