# Supplementary material for: Proteomic Profiling of Bronchoalveolar Lavage Fluid in Critically Ill Patients with Ventilator-Associated Pneumonia
Source: PLoS One. 2013 Mar 7;8(3):e58782. doi: 10.1371/journal.pone.0058782 (PMC3591362; doi:10.1371/journal.pone.0058782)
Supplement: Table S2 — Characteristics of patients suspected of having VAP in the validation cohort. (PDF) [file pone.0058782.s003.pdf]

Table S2. Characteristics of patients suspected of having VAP in the validation cohort.

**Subject characteristics**	**VAP$^+$ (n = 7)**	**VAP$^-$ (n = 8)**	***P*-value†**
Age (mean ± SD)	48 ± 15	58 ± 15	0.22
Gender	7 male, 0 female	7 male, 1 female	
Ventilator days* (mean ± SD)	9 ± 3	10 ± 4	0.67
P_aO_2/F_iO_2** (mean ± SD)	194 ± 43 mmHg	243 ± 45 mmHg	0.052

*Number of days on the ventilator prior to bronchoscopy
**Ratio of arterial oxygen partial pressure (P_aO_2) to fraction of inspired oxygen (F_iO_2) at time of bronchoscopy
†Two-tailed t-test with unequal variance
