# Supplementary material for: Proteomic Profiling of Bronchoalveolar Lavage Fluid in Critically Ill Patients with Ventilator-Associated Pneumonia
Source: PLoS One. 2013 Mar 7;8(3):e58782. doi: 10.1371/journal.pone.0058782 (PMC3591362; doi:10.1371/journal.pone.0058782)
Supplement: Table S3 — BALF proteome of Control subjects with spectral counts. (PDF) [file pone.0058782.s004.pdf]

Table S3. BALF proteome of Control subjects with spectral counts.

Protein IPI	Entrez Gene ID	Gene Symbol	Control1	Control2	Control3	Control4	Control5
IPI00478003	2	A2M	23	27	3	4	29
IPI00550991	12	SERPINA3	9	14	7	6	10
IPI00027442	16	AARS	1	0	1	1	0
IPI00008485	48	ACO1	3	1	1	7	0
IPI00013808	81	ACTN4	2	0	1	3	3
IPI00032220	183	AGT	8	5	4	3	5
IPI00012007	191	AHCY	2	1	1	2	0
IPI00022431	197	AHSG	2	3	1	2	0
IPI00745872	213	ALB	93	50	100	8	14
IPI00218914	216	ALDH1A1	13	8	12	24	22
IPI00479877	223	ALDH9A1	1	0	0	2	1
IPI00465439	226	ALDOA	6	3	0	1	1
IPI00221221	246	ALOX15	5	1	4	3	12
IPI00022426	259	AMBP	2	3	3	0	1
IPI00218918	301	ANXA1	5	4	3	3	2
IPI00024095	306	ANXA3	6	3	0	4	3
IPI00793199	307	ANXA4	6	0	1	5	7
IPI00329801	308	ANXA5	12	6	3	19	12
IPI00414320	311	ANXA11	0	1	4	0	1
IPI00021841	335	APOA1	12	14	21	5	14
IPI00021854	336	APOA2	1	1	2	1	2
IPI00304273	337	APOA4	2	5	12	0	7
IPI00021842	348	APOE	0	2	4	6	4
IPI00218693	353	APRT	2	2	1	1	0
IPI00013698	427	ASAH1	1	1	1	1	0
IPI00032179	462	SERPINC1	6	4	4	3	3
IPI00289499	471	ATIC	2	0	1	3	1
IPI00019591	629	CFB	6	4	5	2	3
IPI00294158	644	BLVRA	3	3	2	5	1
IPI00291866	710	SERPING1	19	1	1	1	1
IPI00022392	712	C1QA	2	2	0	4	0
IPI00477992	713	C1QB	5	3	2	4	3
IPI00022394	714	C1QC	6	3	2	3	2
IPI00783987	718	C3	149	125	148	158	154
IPI00418163	721	C4B	1	1	2	1	1
IPI00021727	722	C4BPA	4	1	7	6	6
IPI00032291	727	C5	7	7	16	14	1
IPI00296608	730	C7	1	1	1	0	0
IPI00294395	732	C8B	0	1	2	1	2
IPI00022395	735	C9	5	3	7	2	2
IPI00011285	823	CAPN1	2	0	4	9	3
IPI00289758	824	CAPN2	3	1	5	4	2
IPI00025084	826	CAPNS1	1	0	3	2	1

IPI00465352	828	CAPS	4	0	5	6	8
IPI00005969	829	CAPZA1	1	0	1	2	0
IPI00026185	832	CAPZB	1	1	2	1	1
IPI00027482	866	SERPINA6	1	2	0	1	1
IPI00295386	873	CBR1	6	4	3	5	0
IPI00027626	908	CCT6A	1	0	1	1	0
IPI00215997	928	CD9	1	0	1	2	1
IPI00418495	948	CD36	1	0	1	1	0
IPI00217766	950	SCARB2	1	0	1	3	0
IPI00297160	960	CD44	4	2	1	3	1
IPI00011302	966	CD59	2	1	1	1	3
IPI00000190	975	CD81	2	1	1	1	2
IPI00016786	998	CDC42	3	2	3	3	1
IPI00010180	1066	CES1	2	2	0	2	0
IPI00012011	1072	CFL1	2	4	1	2	2
IPI00022810	1075	CTSC	1	1	0	1	0
IPI00022977	1152	CKB	3	0	3	3	1
IPI00291262	1191	CLU	5	6	6	3	6
IPI00010896	1192	CLIC1	4	4	2	6	3
IPI00024067	1213	CLTC	4	2	3	12	6
IPI00295851	1315	COPB1	1	0	1	2	1
IPI00017601	1356	CP	19	14	5	13	5
IPI00305477	1469	CST1	1	2	0	0	2
IPI00013382	1470	CST2	2	1	0	0	2
IPI00032294	1472	CST4	5	2	0	0	2
IPI00297487	1512	CTSH	3	1	0	2	1
IPI00152418	1604	CD55	1	2	2	1	1
IPI00005721	1667	DEFA1	12	3	5	3	12
IPI00456969	1778	DYNC1H1	3	1	5	13	5
IPI00257508	1808	DPYSL2	2	0	2	2	0
IPI00292858	1890	TYMP	5	5	3	6	3
IPI00186290	1938	EEF2	7	5	8	9	5
IPI00027444	1992	SERPINB1	9	5	3	8	0
IPI00465248	2023	ENO1	7	5	2	6	2
IPI00219682	2040	STOM	3	1	3	5	3
IPI00019568	2147	F2	3	3	6	0	0
IPI00215746	2167	FABP4	6	3	0	3	0
IPI00029658	2202	EFEMP1	2	1	1	1	2
IPI00073772	2203	FBP1	2	11	6	12	2
IPI00021885	2243	FGA	1	0	2	0	2
IPI00298497	2244	FGB	6	4	16	2	10
IPI00021891	2266	FGG	6	2	12	3	8
IPI00216008	2539	G6PD	2	0	1	0	1
IPI00031461	2665	GDI2	1	1	2	2	1
IPI00215767	2683	B4GALT1	1	0	1	1	1
IPI00217906	2771	GNAI2	2	2	4	5	3

IPI00026268	2782	GNB1	1	0	2	0	1
IPI00295777	2819	GPD1	4	4	6	8	1
IPI00027497	2821	GPI	0	3	4	9	2
IPI00026314	2934	GSN	5	8	4	0	0
IPI00657682	2938	GSTA1	2	2	4	1	5
IPI00219757	2950	GSTP1	13	2	6	9	12
IPI00026272	3012	HIST1H2AE	1	2	1	1	0
IPI00410714	3039	HBA1	4	4	3	0	0
IPI00654755	3043	HBB	5	1	3	0	3
IPI00292950	3053	SERPIND1	3	3	7	3	3
IPI00029739	3075	CFH	1	0	1	1	10
IPI00018246	3098	HK1	2	2	4	7	1
IPI00005118	3101	HK3	5	3	4	11	3
IPI00030023	3176	HNMT	0	1	1	1	0
IPI00216049	3190	HNRNPK	6	2	2	2	0
IPI00022488	3263	HPX	15	17	2	6	3
IPI00022371	3273	HRG	2	3	3	1	2
IPI00304925	3303	HSPA1A	4	0	2	3	0
IPI00003362	3309	HSPA5	3	1	0	1	0
IPI00025512	3315	HSPB1	5	2	1	0	0
IPI00382470	3320	HSP90AA1	13	1	2	23	15
IPI00008494	3383	ICAM1	5	3	3	0	3
IPI00027223	3417	IDH1	5	6	7	5	6
IPI00020996	3483	IGFALS	1	2	1	0	0
IPI00178926	3512	IGJ	3	3	2	3	4
IPI00103356	3689	ITGB2	0	0	2	3	1
IPI00292530	3697	ITIH1	4	7	4	3	4
IPI00305461	3698	ITIH2	4	4	3	0	0
IPI00218192	3700	ITIH4	2	4	3	0	2
IPI00012837	3799	KIF5B	2	0	1	1	0
IPI00001639	3837	KPNB1	1	0	1	3	1
IPI00220327	3848	KRT1	22	8	13	16	14
IPI00019359	3857	KRT9	7	4	6	8	7
IPI00009865	3858	KRT10	2	0	13	1	14
IPI00010471	3936	LCP1	25	29	14	29	8
IPI00219217	3945	LDHB	9	7	7	12	8
IPI00219219	3956	LGALS1	1	0	1	2	0
IPI00023673	3959	LGALS3BP	1	2	1	3	2
IPI00219077	4048	LTA4H	1	1	1	1	1
IPI00298860	4057	LTF	1	1	0	0	1
IPI00019038	4069	LYZ	1	2	0	0	2
IPI00291005	4190	MDH1	7	5	7	9	2
IPI00027848	4360	MRC1	15	16	1	11	5
IPI00219365	4478	MSN	24	19	22	23	12
IPI00019502	4627	MYH9	12	7	11	27	3
IPI00306960	4677	NARS	1	1	0	3	1

IPI00000874	5052	PRDX1	2	2	3	4	3
IPI00016610	5093	PCBP1	1	0	1	1	1
IPI00006114	5176	SERPINF1	7	7	4	4	3
IPI00216691	5216	PFN1	5	6	3	4	3
IPI00219525	5226	PGD	6	5	6	9	8
IPI00169383	5230	PGK1	9	5	6	7	5
IPI00219526	5236	PGM1	5	2	1	0	0
IPI00553177	5265	SERPINA1	15	24	21	1	1
IPI00328609	5267	SERPINA4	1	1	2	0	0
IPI00413451	5269	SERPINB6	6	3	5	5	4
IPI00004573	5284	PIGR	52	33	15	45	32
IPI00022974	5304	PIP	2	2	0	1	4
IPI00847989	5315	PKM2	2	0	4	3	1
IPI00019580	5340	PLG	1	2	1	0	0
IPI00008164	5550	PREP	1	0	1	1	0
IPI00294004	5627	PROS1	1	0	0	1	1
IPI00012503	5660	PSAP	1	1	1	3	1
IPI00479722	5720	PSME1	3	1	1	3	0
IPI00384051	5721	PSME2	3	1	0	2	0
IPI00003590	5768	QSOX1	2	5	0	7	4
IPI00783313	5836	PYGL	3	1	0	5	0
IPI00550069	6050	RNH1	2	6	12	15	8
IPI00642211	6051	RNPEP	5	0	3	8	3
IPI00032313	6275	S100A4	1	1	2	2	1
IPI00027463	6277	S100A6	0	0	6	4	3
IPI00007047	6279	S100A8	4	2	2	3	6
IPI00027462	6280	S100A9	4	1	1	2	3
IPI00013895	6282	S100A11	1	1	3	5	3
IPI00022204	6317	SERPINB3	1	1	0	1	3
IPI00296083	6439	SFTPB	36	27	46	45	22
IPI00291878	6441	SFTPD	6	5	5	3	4
IPI00299095	6643	SNX2	0	1	1	2	0
IPI00030781	6772	STAT1	2	0	2	1	0
IPI00744692	6888	TALDO1	1	2	0	1	2
IPI00292946	6906	SERPINA7	3	1	2	0	0
IPI00022463	7018	TF	43	38	19	14	18
IPI00022462	7037	TFRC	5	3	3	8	1
IPI00018219	7045	TGFBI	6	5	2	2	1
IPI00218251	7052	TGM2	7	5	11	2	4
IPI00643920	7086	TKT	3	2	2	2	1
IPI00298994	7094	TLN1	7	4	8	8	1
IPI00022432	7276	TTR	1	5	6	1	2
IPI00026119	7317	UBA1	4	2	7	8	6
IPI00006705	7356	SCGB1A1	3	0	1	1	0
IPI00031420	7358	UGDH	0	1	1	0	3
IPI00329331	7360	UGP2	2	1	3	3	2

IPI00022774	7415	VCP	9	4	3	9	5
IPI00418471	7431	VIM	3	11	19	53	22
IPI00298971	7448	VTN	3	0	5	1	2
IPI00295400	7453	WARS	3	2	3	7	3
IPI00021263	7534	YWHAZ	1	1	0	1	1
IPI00016342	7879	RAB7A	1	0	2	3	0
IPI00024664	8078	USP5	1	0	0	1	1
IPI00453473	8294	HIST1H4I	2	0	2	2	1
IPI00003935	8349	HIST2H2BE	1	0	2	3	0
IPI00550363	8407	TAGLN2	7	7	1	3	3
IPI00013004	8566	PDXK	0	0	1	3	1
IPI00305978	8574	AKR7A2	0	0	1	1	1
IPI00029012	8661	EIF3A	1	0	0	1	1
IPI00009342	8826	IQGAP1	9	6	9	29	5
IPI00242956	8857	FCGBP	2	8	0	10	2
IPI00012303	8991	SELENBP1	4	1	0	4	1
IPI00020436	9230	RAB11B	1	0	1	2	0
IPI00014055	9476	NAPSA	5	6	4	6	4
IPI00026216	9520	NPEPPS	1	0	1	3	2
IPI00220301	9588	PRDX6	7	4	2	4	3
IPI00000105	9961	MVP	0	0	1	1	1
IPI00246058	10015	PDCD6IP	6	2	6	3	2
IPI00005160	10095	ARPC1B	2	2	2	3	1
IPI00028091	10096	ACTR3	3	4	4	9	2
IPI00030936	10103	TSPAN1	1	0	1	2	2
IPI00005161	10109	ARPC2	1	2	2	6	0
IPI00018873	10135	NAMPT	0	1	1	3	1
IPI00641950	10399	GNB2L1	0	1	2	3	1
IPI00008274	10487	CAP1	2	2	5	0	0
IPI00156689	10493	VAT1	2	5	2	3	0
IPI00018451	10519	CIB1	2	1	2	1	3
IPI00007910	10568	SLC34A2	5	1	1	7	3
IPI00018465	10574	CCT7	1	0	1	1	2
IPI00302927	10575	CCT4	3	0	1	3	2
IPI00297779	10576	CCT2	4	1	1	1	1
IPI00302925	10694	CCT8	2	1	2	1	0
IPI00009104	10856	RUVBL2	1	0	1	1	3
IPI00010720	22948	CCT5	1	0	1	2	1
IPI00017704	23406	COTL1	1	6	1	4	0
IPI00029997	25796	PGLS	2	0	2	2	0
IPI00024915	25824	PRDX5	3	2	2	0	3
IPI00294739	25939	SAMHD1	1	1	2	6	1
IPI00419237	51056	LAP3	5	3	1	2	0
IPI00009856	51297	PLUNC	3	0	0	1	7
IPI00219953	51727	CMPK1	0	1	1	1	2
IPI00147874	54187	NANS	1	0	0	1	1

IPI00451624	55118	CRTAC1	8	8	1	2	3
IPI00296526	55577	NAGK	1	0	2	1	0
IPI00000581	55611	OTUB1	3	0	1	1	1
IPI00018931	55737	VPS35	2	0	2	7	2
IPI00100160	55832	CAND1	4	0	0	3	1
IPI00021302	56241	SUSD2	8	3	4	2	3
IPI00007058	57175	CORO1B	1	2	1	0	0
IPI00456750	64855	FAM129B	2	0	1	0	1
IPI00296654	80341	BPIL1	5	3	1	3	5
IPI00216699	83706	FERMT3	4	2	5	13	1
IPI00066193	92304	SCGB3A1	2	3	3	1	2
IPI00291410	92747	C20orf114	5	28	3	19	45
IPI00296353	93663	ARHGAP18	3	0	3	0	1
IPI00033494	103910	MRLC2	1	0	1	1	0
IPI00163207	114770	PGLYRP2	2	4	1	0	0
IPI00060715	115207	KCTD12	2	0	0	3	1
IPI00328350	116496	FAM129A	3	1	1	3	1
IPI00060800	124220	LOC124220	4	1	0	3	5
IPI00304557	140683	C20orf70	3	1	0	0	4
IPI00166766	146556	MGC45438	2	2	0	0	1
IPI00163563	157310	PEBP4	2	0	2	0	1
IPI00374315	352999	C6orf58	5	2	0	1	6
IPI00012889	653509	SFTPA1	5	3	8	7	4
IPI00855918	727897	MUC5B	45	27	18	45	10
