# Supplementary material for: Proteomic Profiling of Bronchoalveolar Lavage Fluid in Critically Ill Patients with Ventilator-Associated Pneumonia
Source: PLoS One. 2013 Mar 7;8(3):e58782. doi: 10.1371/journal.pone.0058782 (PMC3591362; doi:10.1371/journal.pone.0058782)
Supplement: Table S4 — Differential BALF protein expression between Controls and ALI patients. (PDF) [file pone.0058782.s005.pdf]

Table S4. Differential BALF protein expression between Controls and ALI patients. Significance was based on Spectral Index (SI) analysis. Positive SI indicates increased abundance in ALI whereas negative SI corresponds to increased protein abudance in Control BALF samples.

Protein IPI	Entrez Gene ID	Gene Symbol	Spectral Index	Mean Control Spectral Count	Mean ALI Spectral Count
IPI00021439	60	ACTB	1.000	0.0	174.9
IPI00414676	3326	HSP90AB1	1.000	0.0	11.8
IPI00746388	7430	EZR	1.000	0.0	12.0
IPI00739237	653879	LOC653879	1.000	0.0	96.1
IPI00021885	2243	FGA	0.986	1.0	112.8
IPI00012889	653509	SFTPA1	0.986	5.4	755.1
IPI00013508	87	ACTN1	0.967	0.0	20.9
IPI00215914	375	ARF1	0.967	0.0	3.3
IPI00032258	720	C4A	0.967	0.0	126.9
IPI00219018	2597	GAPDH	0.967	0.0	21.7
IPI00217966	3939	LDHA	0.967	0.0	14.0
IPI00103397	4586	MUC5AC	0.967	0.0	31.1
IPI00789605	4637	MYL6	0.967	0.0	4.7
IPI00552578	6288	SAA1	0.967	0.0	8.2
IPI00099110	1755	DMBT1	0.934	1.0	17.2
IPI00418169	302	ANXA2	0.933	0.0	11.1
IPI00013955	4582	MUC1	0.933	0.0	2.8
IPI00745872	213	ALB	0.932	53.0	1506.7
IPI00027462	6280	S100A9	0.932	2.2	62.4
IPI00003865	3312	HSPA8	0.930	0.6	9.6
IPI00302592	2316	FLNA	0.925	0.2	26.4
IPI00005159	10097	ACTR2	0.900	0.0	8.5
IPI00455174	57186	C20orf74	0.900	0.0	2.9
IPI00166768	84790	TUBA1C	0.900	0.0	1.8
IPI00011654	203068	TUBB	0.900	0.0	4.6
IPI00000816	7531	YWHAE	0.899	0.2	6.4
IPI00219077	4048	LTA4H	0.891	1.0	25.0
IPI00007047	6279	S100A8	0.880	3.4	53.3
IPI00021263	7534	YWHAZ	0.870	0.8	10.3
IPI00022229	338	APOB	0.867	0.0	25.2
IPI00025447	1915	EEF1A1	0.867	0.0	13.3
IPI00216318	7529	YWHAB	0.867	0.0	5.8
IPI00298497	2244	FGB	0.865	7.6	104.6
IPI00010133	11151	CORO1A	0.862	0.4	13.3
IPI00021891	2266	FGG	0.849	6.2	75.8
IPI00298860	4057	LTF	0.846	0.6	41.5
IPI00431645	3240	HP	0.840	0.6	23.1
IPI00783313	5836	PYGL	0.834	1.8	19.5
IPI00179330	6233	RPS27A	0.833	0.0	2.2
IPI00654755	3043	HBB	0.832	2.4	38.7
IPI00643920	7086	TKT	0.826	2.0	21.0
IPI00554811	10093	ARPC4	0.822	0.4	6.2

IPI00555812	2638	GC	0.817	0.4	9.9
IPI00218646	1536	CYBB	0.815	0.4	5.7
IPI00028064	1511	CTSG	0.803	0.2	6.6
IPI00478231	387	RHOA	0.800	0.0	3.5
IPI00021304	3849	KRT2	0.800	0.0	3.9
IPI00022429	5004	ORM1	0.800	0.0	2.7
IPI00027230	7184	HSP90B1	0.800	0.4	4.0
IPI00220642	7532	YWHAG	0.800	0.0	3.1
IPI00797709	23603	CORO1C	0.800	0.0	2.2
IPI00216008	2539	G6PD	0.798	0.8	11.0
IPI00453473	8294	HIST1H4I	0.795	1.4	16.2
IPI00002459	309	ANXA6	0.792	0.4	11.4
IPI00553177	5265	SERPINA1	0.780	12.4	100.5
IPI00003935	8349	HIST2H2BE	0.780	1.2	7.5
IPI00000105	9961	MVP	0.770	0.6	6.3
IPI00013808	81	ACTN4	0.770	1.8	12.3
IPI00168728	3507	IGHM	0.767	0.0	2.3
IPI00217987	3684	ITGAM	0.767	0.0	9.3
IPI00027423	5499	PPP1CA	0.767	0.0	1.3
IPI00171611	126961	HIST2H3C	0.767	0.0	6.6
IPI00410714	3039	HBA1	0.765	2.2	22.2
IPI00019502	4627	MYH9	0.735	12.0	78.5
IPI00004656	567	B2M	0.733	0.0	1.8
IPI00026781	2194	FASN	0.733	0.0	7.4
IPI00032311	3929	LBP	0.733	0.0	9.6
IPI00299024	10409	BASP1	0.733	0.0	2.3
IPI00007244	4353	MPO	0.724	0.6	15.7
IPI00025491	1973	EIF4A1	0.722	0.6	3.8
IPI00419585	5478	PPIA	0.715	0.2	3.5
IPI00478003	2	A2M	0.712	17.2	115.7
IPI00291175	7414	VCL	0.710	0.8	5.7
IPI00291410	92747	C20orf114	0.701	20.0	113.8
IPI00018873	10135	NAMPT	0.686	1.2	7.2
IPI00218918	301	ANXA1	0.672	3.4	19.3
IPI00219038	3020	H3F3A	0.667	0.0	2.7
IPI00216319	7533	YWHAH	0.667	0.0	1.9
IPI00004524	25801	GCA	0.667	0.0	4.0
IPI00298971	7448	VTN	0.657	2.2	11.6
IPI00304925	3303	HSPA1A	0.650	1.8	7.9
IPI00008274	10487	CAP1	0.646	1.8	7.0
IPI00022389	1401	CRP	0.633	0.0	3.2
IPI00027769	1991	ELA2	0.633	0.0	7.2
IPI00027509	4318	MMP9	0.633	0.0	6.7
IPI00010796	5034	P4HB	0.633	0.0	3.9
IPI00019580	5340	PLG	0.631	0.8	9.6
IPI00168184	5518	PPP2R1A	0.627	0.6	3.6
IPI00299547	3934	LCN2	0.617	1.2	4.9
IPI00305461	3698	ITIH2	0.616	2.2	22.8

IPI00026314	2934	GSN	0.612	3.4	11.6
IPI00003817	397	ARHGDIB	0.605	0.2	2.6
IPI00183046	5777	PTPN6	0.605	0.8	4.1
IPI00641229	3494	IGHA2	0.600	0.0	1.9
IPI00255052	4715	NDUFB9	0.600	0.0	0.8
IPI00003909	6515	SLC2A3	0.600	0.0	2.2
IPI00016513	10890	RAB10	0.600	0.0	0.7
IPI00165579	55748	CNDP2	0.600	0.0	3.0
IPI00021854	336	APOA2	0.589	1.4	8.0
IPI00025084	826	CAPNS1	0.588	1.4	5.1
IPI00029739	3075	CFH	0.588	2.6	12.9
IPI00155168	5788	PTPRC	0.577	0.2	2.8
IPI00218319	7170	TPM3	0.576	0.8	2.8
IPI00219682	2040	STOM	0.572	3.0	12.0
IPI00022395	735	C9	0.570	3.8	20.1
IPI00384051	5721	PSME2	0.570	1.2	3.9
IPI00010779	7171	TPM4	0.569	0.2	2.4
IPI00552280	671	BPI	0.567	0.0	6.0
IPI00000875	1937	EEF1G	0.567	0.0	1.2
IPI00892671	3500	IGHG1	0.567	0.0	1.4
IPI00029863	5345	SERPINF2	0.567	0.0	2.2
IPI00293925	8547	FCN3	0.567	0.0	1.5
IPI00012500	83990	BRIP1	0.567	0.0	2.0
IPI00796878	94160	ABCC12	0.567	0.0	5.1
IPI00879709	729	C6	0.563	0.2	4.1
IPI00298994	7094	TLN1	0.561	5.6	23.5
IPI00014338	4689	NCF4	0.558	0.2	1.4
IPI00216256	9948	WDR1	0.556	1.4	4.3
IPI00847989	5315	PKM2	0.554	2.0	7.8
IPI00296353	93663	ARHGAP18	-0.495	1.4	0.2
IPI00294158	644	BLVRA	-0.498	2.8	1.6
IPI00022974	5304	PIP	-0.508	1.8	0.6
IPI00060715	115207	KCTD12	-0.530	1.2	0.1
IPI00022432	7276	TTR	-0.538	3.0	1.5
IPI00000874	5052	PRDX1	-0.541	2.8	1.4
IPI00006114	5176	SERPINF1	-0.543	5.0	2.0
IPI00479877	223	ALDH9A1	-0.549	0.8	0.1
IPI00010180	1066	CES1	-0.549	1.2	0.1
IPI00163563	157310	PEBP4	-0.558	1.0	0.1
IPI00008164	5550	PREP	-0.567	0.6	0.0
IPI00744692	6888	TALDO1	-0.568	1.2	0.3
IPI00031420	7358	UGDH	-0.580	1.0	0.0
IPI00419237	51056	LAP3	-0.596	2.2	0.5
IPI00022810	1075	CTSC	-0.600	0.6	0.0
IPI00305477	1469	CST1	-0.600	1.0	0.0
IPI00013382	1470	CST2	-0.600	1.0	0.0
IPI00032294	1472	CST4	-0.600	1.8	0.0
IPI00257508	1808	DPYSL2	-0.600	1.2	0.0

IPI00215746	2167	FABP4	-0.600	2.4	0.0
IPI00292946	6906	SERPINA7	-0.600	1.2	0.0
IPI00020436	9230	RAB11B	-0.600	0.8	0.0
IPI00304557	140683	C20orf70	-0.600	1.6	0.0
IPI00220301	9588	PRDX6	-0.610	4.0	1.4
IPI00295386	873	CBR1	-0.632	3.6	0.7
IPI00022488	3263	HPX	-0.635	8.6	2.9
IPI00374315	352999	C6orf58	-0.675	2.8	0.4
IPI00291005	4190	MDH1	-0.708	6.0	1.5
IPI00032179	462	SERPINC1	-0.713	4.0	1.0
IPI00008485	48	ACO1	-0.726	2.4	0.2
IPI00017704	23406	COTL1	-0.733	2.4	0.2
IPI00021302	56241	SUSD2	-0.735	4.0	0.9
IPI00018246	3098	HK1	-0.758	3.2	0.7
IPI00009104	10856	RUVBL2	-0.777	1.2	0.0
IPI00027482	866	SERPINA6	-0.800	1.0	0.0
IPI00297487	1512	CTSH	-0.800	1.4	0.0
IPI00215767	2683	B4GALT1	-0.800	0.8	0.0
IPI00012303	8991	SELENBP1	-0.800	2.0	0.0
IPI00302925	10694	CCT8	-0.800	1.2	0.0
IPI00022462	7037	TFRC	-0.805	4.0	0.7
IPI00011302	966	CD59	-0.838	1.6	0.3
IPI00413451	5269	SERPINB6	-0.945	4.6	0.2
IPI00027848	4360	MRC1	-0.962	9.6	0.3
