# Supplementary material for: Proteomic Profiling of Bronchoalveolar Lavage Fluid in Critically Ill Patients with Ventilator-Associated Pneumonia
Source: PLoS One. 2013 Mar 7;8(3):e58782. doi: 10.1371/journal.pone.0058782 (PMC3591362; doi:10.1371/journal.pone.0058782)
Supplement: Table S5 — BALF proteome of ALI patients with and without VAP. (PDF) [file pone.0058782.s006.pdf]

Table S5. BALF proteome of ALI patients with and without VAP. Differentially enriched proteins in VAP(+) patients are highlighted in red and those more abundant in VAP(–) subjects are in blue. Significance was based on Spectral Index (SI) analysis (absolute value cutoff ≥ 0.417).

Protein IPI	Entrez Gene ID	Gene Symbol	Description	SI
IPI00027769	1991	ELA2	elastase 2, neutrophil	0.698
IPI00332371	5211	PFKL	phosphofructokinase, liver	0.692
IPI00022975	241	ALOX5AP	arachidonate 5-lipoxygenase-activating protein	0.662
IPI00020984	821	CANX	calnexin	0.649
IPI00003817	397	ARHGDIB	Rho GDP dissociation inhibitor (GDI) beta	0.648
IPI00007244	4353	MPO	myeloperoxidase	0.627
IPI00552280	671	BPI	bactericidal/permeability-increasing protein	0.625
IPI00218433	1535	CYBA	cytochrome b-245, alpha polypeptide	0.613
IPI00217987	3684	ITGAM	integrin, alpha M (complement component 3 receptor 3 subunit)	0.604
IPI00009856	51297	PLUNC	palate, lung and nasal epithelium associated	0.604
IPI00027509	4318	MMP9	matrix metallopeptidase 9	0.595
IPI00218916	240	ALOX5	arachidonate 5-lipoxygenase	0.586
IPI00103356	3689	ITGB2	integrin, beta 2 (complement component 3 receptor 3 and 4 subunit)	0.584
IPI00025252	2923	PDIA3	protein disulfide isomerase family A, member 3	0.580
IPI00654777	8665	EIF3F	eukaryotic translation initiation factor 3, subunit F	0.578
IPI00007047	6279	S100A8	S100 calcium binding protein A8	0.575
IPI00020210	8291	DYSF	dysferlin, limb girdle muscular dystrophy 2B (autosomal recessive)	0.571
IPI00021926	5706	PSMC6	proteasome (prosome, macropain) 26S subunit, ATPase, 6	0.571
IPI00102685	91663	MYADM	myeloid-associated differentiation marker	0.571
IPI00298860	4057	LTF	lactotransferrin	0.568
IPI00292532	820	CAMP	cathelicidin antimicrobial peptide	0.568
IPI00000875	1937	EEF1G	eukaryotic translation elongation factor 1 gamma	0.554
IPI00221091	6210	RPS15A	ribosomal protein S15a	0.550
IPI00216691	5216	PFN1	profilin 1	0.546
IPI00030872	8875	VNN2	vanin 2	0.543
IPI00003269	345651	ACTBL2	actin, beta-like 2	0.542
IPI00027462	6280	S100A9	S100 calcium binding protein A9	0.540
IPI00783313	5836	PYGL	phosphorylase, glycogen	0.539
IPI00003909	6515	SLC2A3	solute carrier family 2 (facilitated glucose transporter), member 3	0.533
IPI00004524	25801	GCA	grancalcin, EF-hand calcium binding protein	0.530
IPI00027423	5499	PPP1CA	protein phosphatase 1, catalytic subunit, alpha isoform	0.526
IPI00011454	23193	GANAB	glucosidase, alpha; neutral AB	0.524
IPI00295851	1315	COPB1	coatomer protein complex, subunit beta 1	0.521
IPI00296635	2632	GBE1	glucan (1,4-alpha-), branching enzyme 1	0.514
IPI00220219	9276	COPB2	coatomer protein complex, subunit beta 2 (beta prime)	0.504
IPI00020618	8369	HIST1H4G	histone cluster 1, H4g	0.500
IPI00022255	10562	OLFM4	olfactomedin 4	0.497
IPI00027409	5657	PRTN3	proteinase 3	0.496
IPI00007750	7277	TUBA4A	tubulin, alpha 4a	0.485
IPI00016610	5093	PCBP1	poly(rC) binding protein 1	0.483
IPI00012728	2180	ACSL1	acyl-CoA synthetase long-chain family member 1	0.479
IPI00024067	1213	CLTC	clathrin, heavy chain (Hc)	0.474
IPI00013508	87	ACTN1	actinin, alpha 1	0.460
IPI00028064	1511	CTSG	cathepsin G	0.456
IPI00027497	2821	GPI	glucose phosphate isomerase	0.456
IPI00008380	5515	PPP2CA	protein phosphatase 2 (formerly 2A), catalytic subunit, alpha isoform	0.455
IPI00744706	6709	SPTAN1	spectrin, alpha, non-erythrocytic 1 (alpha-fodrin)	0.453
IPI00021290	47	ACLY	ATP citrate lyase	0.451
IPI00413587	637	BID	BH3 interacting domain death agonist	0.450
IPI00010796	5034	P4HB	procollagen-proline, 2-oxoglutarate 4-dioxygenase, beta polypeptide	0.450
IPI00021428	58	ACTA1	actin, alpha 1, skeletal muscle	0.448
IPI00297444	57126	CD177	CD177 molecule	0.446
IPI00242956	8857	FCGBP	Fc fragment of IgG binding protein	0.445
IPI00295857	1314	COPA	coatomer protein complex, subunit alpha	0.445
IPI00155168	5788	PTPRC	protein tyrosine phosphatase, receptor type, C	0.434
IPI00328257	162	AP1B1	adaptor-related protein complex 1, beta 1 subunit	0.430
IPI00017184	10938	EHD1	EH-domain containing 1	0.426

IPI00022391	325	APCS	amyloid P component, serum	0.422
IPI00218638	4542	MYO1F	myosin IF	0.420
IPI00303318	51571	FAM49B	family with sequence similarity 49, member B	0.417
IPI00654755	3043	HBB	hemoglobin, beta	-0.417
IPI00021304	3849	KRT2	keratin 2 (epidermal ichthyosis bullosa of Siemens)	-0.424
IPI00298497	2244	FGB	fibrinogen beta chain	-0.426
IPI00006114	5176	SERPINF1	serpin peptidase inhibitor, clade F , member 1	-0.428
IPI00414283	2335	FN1	fibronectin 1	-0.436
IPI00298828	350	APOH	apolipoprotein H (beta-2-glycoprotein I)	-0.440
IPI00027019	5545	PRB4	proline-rich protein BstNI subfamily 4	-0.453
IPI00796878	94160	ABCC12	ATP-binding cassette, sub-family C (CFTR/MRP), member 12	-0.468
IPI00022895	1	A1BG	alpha-1-B glycoprotein	-0.485
IPI00006705	7356	SCGB1A1	secretoglobin, family 1A, member 1 (uteroglobin)	-0.487
IPI00026320	51366	UBR5	ubiquitin protein ligase E3 component n-recognin 5	-0.516
IPI00009865	3858	KRT10	keratin 10 (epidermolytic hyperkeratosis; keratosis palmaris et plantaris)	-0.522
IPI00028306	85458	DIXDC1	DIX domain containing 1	-0.564
IPI00298237	1200	TPP1	tripeptidyl peptidase I	-0.584
IPI00021885	2243	FGA	fibrinogen alpha chain	-0.589
IPI00216318	7529	YWHAB	tyrosine 3-monooxygenase/tryptophan 5-monooxygenase activation protein, l	0.408
IPI00216699	83706	FERMT3	fermitin family homolog 3 (Drosophila)	0.404
IPI00644712	2547	XRCC6	X-ray repair complementing defective repair in Chinese hamster cells 6	0.400
IPI00306960	4677	NARS	asparaginyl-tRNA synthetase	0.397
IPI00002459	309	ANXA6	annexin A6	0.391
IPI00026185	832	CAPZB	capping protein (actin filament) muscle Z-line, beta	0.391
IPI00004573	5284	PIGR	polymeric immunoglobulin receptor	0.390
IPI00022449	1794	DOCK2	dedicator of cytokinesis 2	0.388
IPI00001639	3837	KPNB1	karyopherin (importin) beta 1	0.387
IPI00297160	960	CD44	CD44 molecule (Indian blood group)	0.384
IPI00296654	80341	BPIL1	bactericidal/permeability-increasing protein-like 1	0.384
IPI00013219	3611	ILK	integrin-linked kinase	0.381
IPI00013163	4332	MNDA	myeloid cell nuclear differentiation antigen	0.378
IPI00783982	22820	COPG	coatomer protein complex, subunit gamma	0.377
IPI00100160	55832	CAND1	cullin-associated and neddylation-dissociated 1	0.377
IPI00026833	159	ADSS	adenylosuccinate synthase	0.377
IPI00299547	3934	LCN2	lipocalin 2	0.376
IPI00014338	4689	NCF4	neutrophil cytosolic factor 4, 40kDa	0.375
IPI00003348	2783	GNB2	guanine nucleotide binding protein (G protein), beta polypeptide 2	0.374
IPI00220834	7520	XRCC5	X-ray repair complementing defective repair in Chinese hamster cells 5	0.374
IPI00029769	3055	HCK	hemopoietic cell kinase	0.373
IPI00025202	752	FMNL1	formin-like 1	0.373
IPI00299024	10409	BASP1	brain abundant, membrane attached signal protein 1	0.372
IPI00027626	908	CCT6A	chaperonin containing TCP1, subunit 6A (zeta 1)	0.370
IPI00019971	6813	STXBP2	syntaxin binding protein 2	0.368
IPI00183046	5777	PTPN6	protein tyrosine phosphatase, non-receptor type 6	0.366
IPI00641229	3494	IGHA2	immunoglobulin heavy constant alpha 2 (A2m marker)	0.365
IPI00218646	1536	CYBB	cytochrome b-245, beta polypeptide (chronic granulomatous disease)	0.365
IPI00060181	79180	EFHD2	EF-hand domain family, member D2	0.364
IPI00216514	961	CD47	CD47 molecule	0.364
IPI00554811	10093	ARPC4	actin related protein 2/3 complex, subunit 4, 20kDa	0.364
IPI00010133	11151	CORO1A	coronin, actin binding protein, 1A	0.363
IPI00005614	6711	SPTBN1	spectrin, beta, non-erythrocytic 1	0.362
IPI00003935	8349	HIST2H2BE	histone cluster 2, H2be	0.360
IPI00031131	57136	C20orf3	chromosome 20 open reading frame 3	0.359
IPI00033494	103910	MRLC2	myosin regulatory light chain MRLC2	0.356
IPI00010270	5880	RAC2	ras-related C3 botulinum toxin substrate 2	0.356
IPI00009342	8826	IQGAP1	IQ motif containing GTPase activating protein 1	0.354
IPI00019038	4069	LYZ	lysozyme (renal amyloidosis)	0.346
IPI00550212	23191	CYFIP1	cytoplasmic FMR1 interacting protein 1	0.345
IPI00018146	10971	YWHAQ	tyrosine 3-monooxygenase/tryptophan 5-monooxygenase activation protein, t	0.345
IPI00029568	5806	PTX3	pentraxin-related gene, rapidly induced by IL-1 beta	0.340
IPI00328350	116496	FAM129A	family with sequence similarity 129, member A	0.338
IPI00020599	811	CALR	calreticulin	0.338

IPI00000105	9961	MVP	major vault protein	0.337
IPI00018465	10574	CCT7	chaperonin containing TCP1, subunit 7 (eta)	0.334
IPI00012578	3840	KPNA4	karyopherin alpha 4 (importin alpha 3)	0.334
IPI00216256	9948	WDR1	WD repeat domain 1	0.330
IPI00302927	10575	CCT4	chaperonin containing TCP1, subunit 4 (delta)	0.330
IPI00643920	7086	TKT	transketolase (Wernicke-Korsakoff syndrome)	0.329
IPI00033022	1785	DNM2	dynamin 2	0.328
IPI00019502	4627	MYH9	myosin, heavy chain 9, non-muscle	0.322
IPI00216008	2539	G6PD	glucose-6-phosphate dehydrogenase	0.317
IPI00005721	1667	DEFA1	defensin, alpha 1; defensin, alpha 1	0.315
IPI00465439	226	ALDOA	aldolase A, fructose-bisphosphate	0.311
IPI00220301	9588	PRDX6	peroxiredoxin 6	0.307
IPI00026268	2782	GNB1	guanine nucleotide binding protein (G protein), beta polypeptide 1	0.304
IPI00294187	11240	PADI2	peptidyl arginine deiminase, type II	0.302
IPI00024915	25824	PRDX5	peroxiredoxin 5	0.299
IPI00552578	6288	SAA1	serum amyloid A1	0.299
IPI00018873	10135	NAMPT	nicotinamide phosphoribosyltransferase	0.299
IPI00152418	1604	CD55	CD55 molecule, decay accelerating factor for complement	0.298
IPI00453473	8294	HIST1H4I	histone cluster 1, H4i	0.293
IPI00218131	6283	S100A12	S100 calcium binding protein A12	0.293
IPI00641950	10399	GNB2L1	guanine nucleotide binding protein (G protein), beta polypeptide 2-like 1	0.292
IPI00021070	4688	NCF2	neutrophil cytosolic factor 2 (65kDa, chronic granulomatous disease, autoson	0.292
IPI00220740	4869	NPM1	nucleophosmin (nucleolar phosphoprotein B23, numatrin)	0.291
IPI00065486	10058	ABCB6	ATP-binding cassette, sub-family B (MDR/TAP), member 6	0.291
IPI00157757	64780	MICAL1	microtubule associated monoxygenase, calponin and LIM domain containing	0.291
IPI00008307	23569	PADI4	peptidyl arginine deiminase, type IV	0.291
IPI00296526	55577	NAGK	N-acetylglucosamine kinase	0.287
IPI00297779	10576	CCT2	chaperonin containing TCP1, subunit 2 (beta)	0.286
IPI00219682	2040	STOM	stomatin	0.284
IPI00012007	191	AHCY	S-adenosylhomocysteine hydrolase	0.279
IPI00171611	126961	HIST2H3C	histone cluster 2, H3c	0.278
IPI00292530	3697	ITIH1	inter-alpha (globulin) inhibitor H1	0.273
IPI00298961	7514	XPO1	exportin 1 (CRM1 homolog, yeast)	0.271
IPI00021263	7534	YWHAZ	tyrosine 3-monooxygenase/tryptophan 5-monooxygenase activation protein, :	0.268
IPI00456969	1778	DYNC1H1	dynein, cytoplasmic 1, heavy chain 1	0.268
IPI00013808	81	ACTN4	actinin, alpha 4	0.267
IPI00003949	7334	UBE2N	ubiquitin-conjugating enzyme E2N (UBC13 homolog, yeast)	0.266
IPI00789605	4637	MYL6	myosin, light chain 6, alkali, smooth muscle and non-muscle	0.266
IPI00024095	306	ANXA3	annexin A3	0.264
IPI00306436	6774	STAT3	signal transducer and activator of transcription 3 (acute-phase response facto	0.263
IPI00005118	3101	HK3	hexokinase 3 (white cell)	0.258
IPI00011285	823	CAPN1	calpain 1, (mu/I) large subunit	0.257
IPI00022977	1152	CKB	creatine kinase, brain	0.256
IPI00027444	1992	SERPINB1	serpin peptidase inhibitor, clade B (ovalbumin), member 1	0.255
IPI00298994	7094	TLN1	talin 1	0.255
IPI00027230	7184	HSP90B1	heat shock protein 90kDa beta (Grp94), member 1	0.254
IPI00147874	54187	NANS	N-acetylneuraminic acid synthase (sialic acid synthase)	0.253
IPI00005162	10094	ARPC3	actin related protein 2/3 complex, subunit 3, 21kDa	0.253
IPI00027007	648998	LOC648998	similar to Neutrophil cytosol factor 1	0.252
IPI00219038	3020	H3F3A	H3 histone, family 3A	0.252
IPI00005159	10097	ACTR2	ARP2 actin-related protein 2 homolog (yeast)	0.250
IPI00220578	2773	GNAI3	guanine nucleotide binding protein (G protein), alpha inhibiting activity polype	0.247
IPI00217906	2771	GNAI2	guanine nucleotide binding protein (G protein), alpha inhibiting activity polype	0.246
IPI00184996	57119	SPINLW1	serine peptidase inhibitor-like, with Kunitz and WAP domains 1 (eppin)	0.246
IPI00022744	1434	CSE1L	CSE1 chromosome segregation 1-like (yeast)	0.244
IPI00007682	523	ATP6V1A	ATPase, H+ transporting, lysosomal 70kDa, V1 subunit A	0.243
IPI00419916	249	ALPL	alkaline phosphatase, liver/bone/kidney	0.240
IPI00021439	60	ACTB	actin, beta	0.238
IPI00010471	3936	LCP1	lymphocyte cytosolic protein 1 (L-plastin)	0.237
IPI00005969	829	CAPZA1	capping protein (actin filament) muscle Z-line, alpha 1	0.236
IPI00384051	5721	PSME2	proteasome (prosome, macropain) activator subunit 2 (PA28 beta)	0.235
IPI00168184	5518	PPP2R1A	protein phosphatase 2 (formerly 2A), regulatory subunit A , alpha isoform	0.229

IPI00292858	1890	TYMP	thymidine phosphorylase	0.228
IPI00217966	3939	LDHA	lactate dehydrogenase A	0.226
IPI00642211	6051	RNPEP	arginyl aminopeptidase (aminopeptidase B)	0.225
IPI00026216	9520	NPEPPS	aminopeptidase puromycin sensitive	0.225
IPI00022229	338	APOB	apolipoprotein B (including Ag(x) antigen)	0.222
IPI00028091	10096	ACTR3	ARP3 actin-related protein 3 homolog (yeast)	0.222
IPI00005161	10109	ARPC2	actin related protein 2/3 complex, subunit 2, 34kDa	0.221
IPI00013418	329	BIRC2	baculoviral IAP repeat-containing 2	0.220
IPI00018931	55737	VPS35	vacuolar protein sorting 35 homolog (S. cerevisiae)	0.208
IPI00022774	7415	VCP	valosin-containing protein	0.204
IPI00000873	7407	VARS	valyl-tRNA synthetase	0.203
IPI00026781	2194	FASN	fatty acid synthase	0.202
IPI00029468	10121	ACTR1A	ARP1 actin-related protein 1 homolog A, centractin alpha (yeast)	0.195
IPI00011654	203068	TUBB	tubulin, beta	0.190
IPI00329331	7360	UGP2	UDP-glucose pyrophosphorylase 2	0.185
IPI00855918	727897	MUC5B	mucin 5B, oligomeric mucus/gel-forming	0.181
IPI00026087	8815	BANF1	barrier to autointegration factor 1	0.178
IPI00022371	3273	HRG	histidine-rich glycoprotein	0.178
IPI00103552	94025	MUC16	mucin 16, cell surface associated	0.175
IPI00000816	7531	YWHAE	tyrosine 3-monooxygenase/tryptophan 5-monooxygenase activation protein, (	0.168
IPI00010896	1192	CLIC1	chloride intracellular channel 1	0.168
IPI00219077	4048	LTA4H	leukotriene A4 hydrolase	0.168
IPI00020567	392	ARHGAP1	Rho GTPase activating protein 1	0.163
IPI00477597	3250	HPR	haptoglobin-related protein	0.159
IPI00641073	80736	SLC44A4	solute carrier family 44, member 4	0.158
IPI00004358	5834	PYGB	phosphorylase, glycogen; brain	0.156
IPI00296608	730	C7	complement component 7	0.142
IPI00026119	7317	UBA1	ubiquitin-like modifier activating enzyme 1	0.141
IPI00018342	203	AK1	adenylate kinase 1	0.139
IPI00739237	653879	LOC653879	similar to Complement C3 precursor	0.138
IPI00465248	2023	ENO1	enolase 1, (alpha)	0.137
IPI00003865	3312	HSPA8	heat shock 70kDa protein 8	0.137
IPI00000874	5052	PRDX1	peroxiredoxin 1	0.131
IPI00012500	83990	BRIP1	BRCA1 interacting protein C-terminal helicase 1	0.128
IPI00218993	10808	HSPH1	heat shock 105kDa/110kDa protein 1	0.125
IPI00023860	4673	NAP1L1	nucleosome assembly protein 1-like 1	0.124
IPI00219365	4478	MSN	moesin	0.122
IPI00008433	6193	RPS5	ribosomal protein S5	0.122
IPI00257882	5184	PEPD	peptidase D	0.121
IPI00290770	7203	CCT3	chaperonin containing TCP1, subunit 3 (gamma)	0.121
IPI00294739	25939	SAMHD1	SAM domain and HD domain 1	0.120
IPI00012503	5660	PSAP	prosaposin (variant Gaucher disease and variant metachromatic leukodystrop	0.118
IPI00030936	10103	TSPAN1	tetraspanin 1	0.109
IPI00305461	3698	ITIH2	inter-alpha (globulin) inhibitor H2	0.107
IPI00219525	5226	PGD	phosphogluconate dehydrogenase	0.106
IPI00305978	8574	AKR7A2	aldo-keto reductase family 7, member A2 (aflatoxin aldehyde reductase)	0.101
IPI00022426	259	AMBP	alpha-1-microglobulin/bikunin precursor	0.099
IPI00018451	10519	CIB1	calcium and integrin binding 1 (calmyrin)	0.098
IPI00291175	7414	VCL	vinculin	0.097
IPI00419585	5478	PPIA	peptidylprolyl isomerase A (cyclophilin A)	0.093
IPI00013955	4582	MUC1	mucin 1, cell surface associated	0.090
IPI00550069	6050	RNH1	ribonuclease/angiogenin inhibitor 1	0.089
IPI00643041	5901	RAN	RAN, member RAS oncogene family	0.088
IPI00013004	8566	PDXK	pyridoxal (pyridoxine, vitamin B6) kinase	0.088
IPI00215914	375	ARF1	ADP-ribosylation factor 1	0.086
IPI00027223	3417	IDH1	isocitrate dehydrogenase 1 (NADP+), soluble	0.084
IPI00302592	2316	FLNA	filamin A, alpha (actin binding protein 280)	0.082
IPI00007058	57175	CORO1B	coronin, actin binding protein, 1B	0.081
IPI00103397	4586	MUC5AC	mucin 5AC, oligomeric mucus/gel-forming	0.081
IPI00016513	10890	RAB10	RAB10, member RAS oncogene family	0.080
IPI00418471	7431	VIM	vimentin	0.080
IPI00847989	5315	PKM2	pyruvate kinase, muscle	0.072

IPI00026314	2934	GSN	gelsolin (amyloidosis, Finnish type)	0.071
IPI00023673	3959	LGALS3BP	lectin, galactoside-binding, soluble, 3 binding protein	0.070
IPI00414676	3326	HSP90AB1	heat shock protein 90kDa alpha (cytosolic), class B member 1	0.069
IPI00216319	7533	YWHAH	tyrosine 3-monooxygenase/tryptophan 5-monooxygenase activation protein,	0.067
IPI00030781	6772	STAT1	signal transducer and activator of transcription 1, 91kDa	0.064
IPI00012011	1072	CFL1	cofilin 1 (non-muscle)	0.056
IPI00220642	7532	YWHAG	tyrosine 3-monooxygenase/tryptophan 5-monooxygenase activation protein,	0.055
IPI00007812	526	ATP6V1B2	ATPase, H+ transporting, lysosomal 56/58kDa, V1 subunit B2	0.055
IPI00215768	2729	GCLC	glutamate-cysteine ligase, catalytic subunit	0.055
IPI00000581	55611	OTUB1	OTU domain, ubiquitin aldehyde binding 1	0.051
IPI00745872	213	ALB	albumin	0.051
IPI00178926	3512	IGJ	immunoglobulin J polypeptide, linker protein for immunoglobulin alpha and m	0.046
IPI00291410	92747	C20orf114	chromosome 20 open reading frame 114	0.042
IPI00022389	1401	CRP	C-reactive protein, pentraxin-related	0.040
IPI00027463	6277	S100A6	S100 calcium binding protein A6	0.039
IPI00017601	1356	CP	ceruloplasmin (ferroxidase)	0.038
IPI00165579	55748	CNDP2	CNDP dipeptidase 2 (metallopeptidase M20 family)	0.037
IPI00012889	653509	SFTPA1	surfactant, pulmonary-associated protein A1	0.036
IPI00179330	6233	RPS27A	ribosomal protein S27a	0.036
IPI00298971	7448	VTN	vitronectin	0.035
IPI00431645	3240	HP	haptoglobin	0.034
IPI00186290	1938	EEF2	eukaryotic translation elongation factor 2	0.032
IPI00171199	5684	PSMA3	proteasome (prosome, macropain) subunit, alpha type, 3	0.031
IPI00016786	998	CDC42	cell division cycle 42 (GTP binding protein, 25kDa)	0.029
IPI00219217	3945	LDHB	lactate dehydrogenase B	0.026
IPI00003362	3309	HSPA5	heat shock 70kDa protein 5 (glucose-regulated protein, 78kDa)	0.024
IPI00021854	336	APOA2	apolipoprotein A-II	0.024
IPI00013698	427	ASAH1	N-acylsphingosine amidohydrolase (acid ceramidase) 1	0.022
IPI00032328	3827	KNG1	kininogen 1	0.021
IPI00031461	2665	GDI2	GDP dissociation inhibitor 2	0.016
IPI00246058	10015	PDCD6IP	programmed cell death 6 interacting protein	0.015
IPI00304925	3303	HSPA1A	heat shock 70kDa protein 1A	0.014
IPI00414320	311	ANXA11	annexin A11	0.012
IPI00008274	10487	CAP1	CAP, adenylate cyclase-associated protein 1 (yeast)	0.011
IPI00010720	22948	CCT5	chaperonin containing TCP1, subunit 5 (epsilon)	0.004
IPI00025491	1973	EIF4A1	eukaryotic translation initiation factor 4A, isoform 1	0.004
IPI00018909	7033	TFF3	trefoil factor 3 (intestinal)	0.003
IPI00291262	1191	CLU	clusterin	0.001
IPI00013890	2810	SFN	stratifin	-0.002
IPI00746388	7430	EZR	ezrin	-0.003
IPI00456750	64855	FAM129B	family with sequence similarity 129, member B	-0.005
IPI00025084	826	CAPNS1	calpain, small subunit 1	-0.006
IPI00291866	710	SERPING1	serpin peptidase inhibitor, clade G (C1 inhibitor), member 1, (angioedema, he	-0.018
IPI00005171	3122	HLA-DRA	major histocompatibility complex, class II, DR alpha	-0.018
IPI00026260	4831	NME2	non-metastatic cells 2, protein (NM23B) expressed in	-0.019
IPI00013895	6282	S100A11	S100 calcium binding protein A11	-0.020
IPI00169383	5230	PGK1	phosphoglycerate kinase 1	-0.021
IPI00478231	387	RHOA	ras homolog gene family, member A	-0.022
IPI00032313	6275	S100A4	S100 calcium binding protein A4	-0.026
IPI00218918	301	ANXA1	annexin A1	-0.027
IPI00218251	7052	TGM2	transglutaminase 2 (C polypeptide, protein-glutamine-gamma-glutamyltransfe	-0.027
IPI00218319	7170	TPM3	tropomyosin 3	-0.028
IPI00029260	929	CD14	CD14 molecule	-0.028
IPI00299086	6386	SDCBP	syndecan binding protein (syntenin)	-0.031
IPI00166768	84790	TUBA1C	tubulin, alpha 1c	-0.031
IPI00010779	7171	TPM4	tropomyosin 4	-0.034
IPI00304273	337	APOA4	apolipoprotein A-IV	-0.034
IPI00018031	221	ALDH3B1	aldehyde dehydrogenase 3 family, member B1	-0.038
IPI00215997	928	CD9	CD9 molecule	-0.044
IPI00382470	3320	HSP90AA1	heat shock protein 90kDa alpha (cytosolic), class A member 1	-0.048
IPI00289758	824	CAPN2	calpain 2, (m/II) large subunit	-0.049
IPI00255052	4715	NDUFB9	NADH dehydrogenase (ubiquinone) 1 beta subcomplex, 9, 22kDa	-0.052

IPI00021841	335	APOA1	apolipoprotein A-I	-0.056
IPI00296083	6439	SFTPB	surfactant, pulmonary-associated protein B	-0.060
IPI00156689	10493	VAT1	vesicle amine transport protein 1 homolog (T. californica)	-0.060
IPI00019568	2147	F2	coagulation factor II (thrombin)	-0.061
IPI00022395	735	C9	complement component 9	-0.062
IPI00168728	3507	IGHM	immunoglobulin heavy constant mu	-0.064
IPI00219757	2950	GSTP1	glutathione S-transferase pi	-0.064
IPI00797709	23603	CORO1C	coronin, actin binding protein, 1C	-0.064
IPI00218192	3700	ITIH4	inter-alpha (globulin) inhibitor H4 (plasma Kallikrein-sensitive glycoprotein)	-0.067
IPI00027442	16	AARS	alanyl-tRNA synthetase	-0.067
IPI00032179	462	SERPINC1	serpin peptidase inhibitor, clade C (antithrombin), member 1	-0.071
IPI00000190	975	CD81	CD81 molecule	-0.072
IPI00657682	2938	GSTA1	glutathione S-transferase A1	-0.074
IPI00005160	10095	ARPC1B	actin related protein 2/3 complex, subunit 1B, 41kDa	-0.079
IPI00219018	2597	GAPDH	glyceraldehyde-3-phosphate dehydrogenase	-0.087
IPI00029863	5345	SERPINF2	serpin peptidase inhibitor, clade F, member 2	-0.092
IPI00021857	345	APOC3	apolipoprotein C-III	-0.092
IPI00218732	5444	PON1	paraoxonase 1	-0.094
IPI00555812	2638	GC	group-specific component (vitamin D binding protein)	-0.094
IPI00166866	3493	IGHA1	immunoglobulin heavy constant alpha 1	-0.097
IPI00007067	152007	C9orf19	chromosome 9 open reading frame 19	-0.104
IPI00022429	5004	ORM1	orosomucoid 1	-0.109
IPI00022624	9052	GPRC5A	G protein-coupled receptor, family C, group 5, member A	-0.112
IPI00290566	6950	TCP1	t-complex 1	-0.117
IPI00784002	26278	SACS	spastic ataxia of Charlevoix-Saguenay (sacsin)	-0.119
IPI00006662	347	APOD	apolipoprotein D	-0.125
IPI00478003	2	A2M	alpha-2-macroglobulin	-0.126
IPI00305622	7051	TGM1	transglutaminase 1	-0.129
IPI00032220	183	AGT	angiotensinogen (serpin peptidase inhibitor, clade A, member 8)	-0.133
IPI00418169	302	ANXA2	annexin A2	-0.133
IPI00022488	3263	HPX	hemopexin	-0.134
IPI00099110	1755	DMBT1	deleted in malignant brain tumors 1	-0.136
IPI00292950	3053	SERPIND1	serpin peptidase inhibitor, clade D (heparin cofactor), member 1	-0.137
IPI00019591	629	CFB	complement factor B	-0.149
IPI00027341	822	CAPG	capping protein (actin filament), gelsolin-like	-0.153
IPI00879709	729	C6	complement component 6	-0.153
IPI00793199	307	ANXA4	annexin A4	-0.153
IPI00514026	375790	AGRN	agrin	-0.165
IPI00025447	1915	EEF1A1	eukaryotic translation elongation factor 1 alpha 1	-0.169
IPI00014055	9476	NAPSA	napsin A aspartic peptidase	-0.171
IPI00892671	3500	IGHG1	immunoglobulin heavy constant gamma 1 (G1m marker)	-0.172
IPI00218914	216	ALDH1A1	aldehyde dehydrogenase 1 family, member A1	-0.174
IPI00465352	828	CAPS	calcyphosine	-0.180
IPI00029658	2202	EFEMP1	EGF-containing fibulin-like extracellular matrix protein 1	-0.181
IPI00221221	246	ALOX15	arachidonate 15-lipoxygenase	-0.181
IPI00295400	7453	WARS	tryptophanyl-tRNA synthetase	-0.182
IPI00410714	3039	HBA1	hemoglobin, alpha 1	-0.183
IPI00019580	5340	PLG	plasminogen	-0.184
IPI00296183	218	ALDH3A1	aldehyde dehydrogenase 3 family, memberA1	-0.188
IPI00021855	341	APOC1	apolipoprotein C-I	-0.188
IPI00030706	10598	AHSA1	AHA1, activator of heat shock 90kDa protein ATPase homolog 1 (yeast)	-0.190
IPI00221224	290	ANPEP	alanyl (membrane) aminopeptidase	-0.190
IPI00032258	720	C4A	complement component 4A (Rodgers blood group)	-0.192
IPI00008494	3383	ICAM1	intercellular adhesion molecule 1 (CD54), human rhinovirus receptor	-0.200
IPI00455174	57186	C20orf74	chromosome 20 open reading frame 74	-0.207
IPI00011229	1509	CTSD	cathepsin D	-0.208
IPI00022463	7018	TF	transferrin	-0.221
IPI00032291	727	C5	complement component 5	-0.223
IPI00002966	3308	HSPA4	heat shock 70kDa protein 4	-0.224
IPI00001699	29108	PYCARD	PYD and CARD domain containing	-0.227
IPI00021727	722	C4BPA	complement component 4 binding protein, alpha	-0.230
IPI00553177	5265	SERPINA1	serpin peptidase inhibitor, clade A (alpha-1 antiproteinase, antitrypsin), memb	-0.231

IPI00066193	92304	SCGB3A1	secretoglobin, family 3A, member 1	-0.236
IPI00022394	714	C1QC	complement component 1, q subcomponent, C chain	-0.237
IPI00477992	713	C1QB	complement component 1, q subcomponent, B chain	-0.238
IPI00032311	3929	LBP	lipopolysaccharide binding protein	-0.239
IPI00294395	732	C8B	complement component 8, beta polypeptide	-0.242
IPI00021842	348	APOE	apolipoprotein E	-0.258
IPI00329801	308	ANXA5	annexin A5	-0.258
IPI00004656	567	B2M	beta-2-microglobulin	-0.265
IPI00289499	471	ATIC	5-aminoimidazole-4-carboxamide ribonucleotide formyltransferase/IMP cyclol	-0.268
IPI00022431	197	AHSG	alpha-2-HS-glycoprotein	-0.268
IPI00291878	6441	SFTPD	surfactant, pulmonary-associated protein D	-0.279
IPI00021891	2266	FGG	fibrinogen gamma chain	-0.284
IPI00007910	10568	SLC34A2	solute carrier family 34 (sodium phosphate), member 2	-0.286
IPI00060800	124220	LOC124220	similar to common salivary protein 1	-0.288
IPI00018219	7045	TGFBI	transforming growth factor, beta-induced, 68kDa	-0.304
IPI00478209	401409	RAB19	RAB19, member RAS oncogene family	-0.305
IPI00301618	9147	SDCCAG1	serologically defined colon cancer antigen 1	-0.307
IPI00029739	3075	CFH	complement factor H	-0.309
IPI00220151	9990	SLC12A6	solute carrier family 12 (potassium/chloride transporters), member 6	-0.315
IPI00217778	5360	PLTP	phospholipid transfer protein	-0.317
IPI00073772	2203	FBP1	fructose-1,6-bisphosphatase 1	-0.326
IPI00019359	3857	KRT9	keratin 9 (epidermolytic palmoplantar keratoderma)	-0.350
IPI00020091	5005	ORM2	orosomucoid 2	-0.354
IPI00550363	8407	TAGLN2	transgelin 2	-0.370
IPI00293925	8547	FCN3	ficolin (collagen/fibrinogen domain containing) 3 (Hakata antigen)	-0.385
IPI00220327	3848	KRT1	keratin 1 (epidermolytic hyperkeratosis)	-0.408
IPI00550991	12	SERPINA3	serpin peptidase inhibitor, clade A (alpha-1 antiproteinase, antitrypsin), memb	-0.415
